# Knowledge and practice of healthy behaviors for dementia and stroke prevention in a United States cohort

**DOI:** 10.1038/s41598-025-99246-8

**Published:** 2025-04-30

**Authors:** Jasper R. Senff, Reinier W. P. Tack, Benjamin Y. Q. Tan, Savvina Prapiadou, Tamara N. Kimball, Sharon Ng, Jonathan Duskin, Mark Jun Shah-Ostrowski, Courtney Nunley, H. Bart Brouwers, Zeina Chemali, Gregory Fricchione, Rudolph E. Tanzi, Koen Pouwels, Jonathan Rosand, Nirupama Yechoor, Christopher D. Anderson, Sanjula D. Singh

**Affiliations:** 1 Brain Care Labs, Massachusetts General Hospital, Boston, MA USA; 2 Department of Neurology, Massachusetts General Hospital, Boston, MA USA; 3 Broad Institute of MIT and Harvard, Cambridge, MA USA; 4 Center for Genomic Medicine, Massachusetts General Hospital, Boston, MA USA; 5 Department of Neurology and Neurosurgery, Brain Center Rudolf Magnus, University Medical Center Utrecht, Utrecht, The Netherlands; 6 Division of Neurology, Department of Medicine, National University Hospital, Singapore, Singapore; 7 Department of Neurology, Brigham and Women’s Hospital, Boston, MA USA; 8 Nuffield Department of Population Health, University of Oxford, Oxford, UK; 9 Department of Psychiatry, Massachusetts General Hospital, Boston, MA USA; 10 Benson-Henry Institute for Mind Body Medicine, Massachusetts General Hospital, Boston, MA USA; 11 McCance Center for Brain Health, Massachusetts General Hospital, Harvard Medical School, Revolution Drive 399, Sommerville, MA 02145 USA; 12 Department of Neurosurgery, Elisabeth-TweeSteden Hospital, Tilburg, The Netherlands

**Keywords:** Knowledge, Practice, Healthy behaviors, Dementia, Stroke, Prevention, Neurology, Risk factors, Public health

## Abstract

At least 45% of dementia and 60% of stroke cases are due to modifiable risk factors and could in part be prevented through healthy behavior. This cross-sectional study clustered and characterized a U.S. cohort’s knowledge and practice of healthy behavior associated with dementia and stroke. A total of 1,478 participants (mean age: 45.5 years, 51.8% female) were included. A hierarchical cluster analysis was performed to identify clusters based on the level of knowledge and practice of healthy behavior. We defined knowledge as recognizing eight modifiable risk factors (alcohol, diet, smoking, physical activity, sleep, stress, social relationships, and purpose in life) as important. We defined practice as complying with validated recommendations for each healthy behavior. Three clusters emerged: (I) high knowledge and poor practice (II) high knowledge and good practice, and (III) lower knowledge and poor practice. Participants in the high knowledge and good practice cluster were statistically significantly older, more educated, perceived fewer barriers (financial and time limitations), and more facilitators (motivation or knowing someone with dementia or stroke) compared to the other clusters. Our findings could assist in tailoring preventative strategies to enhance knowledge, translating knowledge into practice, and addressing particular facilitators and barriers per identified cluster.

**Supplementary Information:**

The online version contains supplementary material available at 10.1038/s41598-025-99246-8.

## Introduction

Age-related brain diseases, such as dementia and stroke, are major contributors to global morbidity and mortality. These conditions pose significant challenges to public health, affecting millions of people worldwide^1^. Research suggests that a substantial proportion of dementia cases (45%) and strokes (at least 60%) are due to modifiable risk factors, indicating that they could potentially be prevented by improving these risk factors^2,3^.

Overlapping risk factors for dementia and stroke include alcohol consumption, poor dietary habits, physical inactivity, smoking, inadequate sleep, high stress levels, poor social relationships, and a lack of purpose in life^4^. Addressing these risk factors through behavioral changes is recognized as a key strategy to reduce the burden of dementia and stroke. International efforts are underway to enhance brain health and empower individuals to take proactive care of their brain through risk factor reduction^3,5^. Despite these efforts, the global prevalence of these risk factors is increasing, illustrating the challenge of modifying behavior^6,7^.

Theoretical frameworks propose that effective behavioral change necessitates capability, opportunity, and motivation; individuals need to have the capability (knowledge and skills) to recognize and implement lifestyle modifications that can prevent diseases like dementia and stroke, have access to the necessary resources and supportive environments (opportunity), and possess strong motivation to change behavior^8^. Previous studies have demonstrated that different populations have different levels of capability, opportunity, and motivation with subsequent different barriers and facilitators^9–12^. Behavioral science has demonstrated that knowledge on the importance of modifiable risk factors for the prevent of disease does not necessarily lead to health-related behaviors that reflect this knowledge, with a variety of barriers proposed to explain this discrepancy. Prior research from various countries highlight the importance of differences in knowledge levels and the presence of diverse barriers and facilitators, including both personal (e.g., lack of knowledge or motivation) and structural barriers (e.g., financial or environmental constraints) to achieve behavioral change for dementia and stroke risk reduction^10,11,13,14^. Previous data highlight the necessity in adequately characterizing subgroups, or target populations to achieve behavior change in dementia and stroke risk reduction^13,15^. Understanding the facilitators and barriers of engaging in healthy behavior across different populations can help to develop tailored interventions to achieve sustained behavioral change and healthier lifestyles – ultimately, to lower risks of dementia and stroke.

We therefore aimed to cluster and characterize subgroups of a U.S. Cohort based on their knowledge and practice of healthy behavior associated with dementia and stroke and assess subsequent facilitators and barriers.

## Methods

### Study and survey design

This cross-sectional study included participants aged 18 years or older through the online vendor platform Prolific^16^. We requested a representative sample of the U.S. All participants completed the Harvard-Oxford Brain Care Awareness Survey (HOBCAS) in September 2023. The HOBCAS was developed using validated questionnaires to address perceptions of brain health and age-related brain disease. In short, the survey included demographic, knowledge, motivation, and health-related behavior questions. The survey utilized questions from the ‘*American Community Survey*’^17^ for demographic information. Knowledge questions were sourced from the *‘Stroke Knowledge Assessment Tool’* (SKAT)^18^ and the *‘Dementia Knowledge Assessment Scale’* (DKAS)^19^. To assess motivation, the ‘*Motivation to Change Lifestyle and Health Behaviors for Dementia Risk Reduction Scale’*^20^ was employed. Lastly, the Brain Care Score was used to assess health behaviors, a validated tool that includes 12 modifiable risk factors associated with dementia and stroke^5,21–23^. The complete survey is presented in Table S1.

### Outcomes

The primary outcomes of interest in this study are *(i)* the knowledge that eight modifiable risk factors contribute to keeping the brain healthy and *(ii)* the corresponding practice of healthy behaviors for each factor. The eight modifiable risk factors include *(a)* alcohol intake, *(b)* diet, *(c)* smoking, *(d)* physical activity, *(e)* sleep, *(f)* stress, *(g)* social relationships, and *(h)* purpose in life.

#### Knowledge

To define knowledge, participants were asked to recognize how useful each of the eight factors were for maintaining brain health (Question 43 Table S1). Responses of “somewhat useful” or “very useful” were categorized as knowing the factor to contribute to brain health. In contrast, responses of “not sure”, “not very useful”, and “not useful at all” were categorized as not knowing the factor as a contributor to brain health.

#### Practice of healthy behaviors

Healty behavior was determined by participants’ BCS responses^5,21–23^. For each of the eight risk factors, individuals who scored in the highest category, indicating optimal brain care, were defined as good practice of healthy behavior, while all others were considered to have poor practice of healthy behavior. The criteria for good practice of healthy behavior were: (I) alcohol intake of 0–1 alcoholic drinks per week; (II) never smoked or quit more than a year ago; (III) typical diet following at least 3 out of 5 diet recommendation ( [a] 4.5 servings of fruits or vegetables per day, [b] 2 servings of lean protein per day, [c] ≥ 3 servings of whole grains per day, [d] < 1,500 mg of sodium per day, [e] Less than 36 oz of sugar sweet beverages per week), (IV) engaging in ≥ 150 min of moderate or ≥ 75 min of high-intensity physical activity per week; (V) achieving 6–8 hrs of sleep without sleep problems; (VI) maintaining manageable stress levels that rarely make it difficult to function; (VII) having at least two people, other than a partner or child, whom they feel close to and can talk to about private matters or call upon for help; (VIII) and generally feeling that life has meaning (Q53-57 Table S1).

### Facilitators and barriers

We included several potential facilitators and barriers to knowledge and practice of healthy behaviors based on prior literature^10,11^. The assessed facilitators included the items ‘*I have ever known someone who has had a diagnosis of dementia or stroke*’ (Q13), ‘ *I have ever been a caregiver for someone who has had a diagnosis of dementia or stroke’* (Q14), ‘*I feel at high risk of developing dementia’* (Q31), ‘*I feel at high risk of having a stroke*’ (Q32), ‘*Learning more motivates me to change my lifestyle*’ (Q40), ‘*Having risk factors makes me want to change my lifestyle*’ (Q41), and ‘*I am confident I could change my lifestyle to reduce the risk of dementia and stroke*’ (Q42). The assessed barriers included the items: ‘*Changing lifestyle is difficult over a long period of time’* (Q37), ‘*I am too busy to change my lifestyle’* (Q38), and *‘My financial situation does not allow me to change my lifestyle’* (Q39). Participants answering “somewhat agree” or “strongly agree” to one of the aforementioned statements were categorized as agreeing with this statement, while those answering “not sure”, “somewhat disagree”, and “strongly disagree” were categorized as not agreeing with this statement.

### Statistical analysis

Discrete variables were described as counts and proportions, continuous variables were described as means and standard deviations (S.D.) or medians and interquartile ranges (IQR), as appropriate. This study focused exclusively on analyzing questions from the HOBCAS survey (Table S1) relevant to the current research objectives.

### Cluster analysis

We performed a cluster analysis to identify subgroups of participants with specific behavioral profiles, as defined by the combination of knowledge and practice of health-related behaviors. Hierarchical clustering analysis was performed including the eight knowledge and corresponding eight practices of healthy behavior variables. We included only those participants with complete data on all 16 variables. We used Jaccard distance and inner squared distance (minimum variance algorithm) for hierarchical clustering to compute the distance between clusters^24^. The clustering results were visualized using a dendrogram. We used a total-within-cluster sum of square (WSS) plot to seed the number of clusters, confirmed by the silhouette criterion^25^. Further, to evaluate the stability of the identified clusters, we performed a cluster-wise Jaccard bootstrap analysis by resampling the data 100 times and calculating the Jaccard bootstrap mean for each cluster^24^. We visualized the clusters using a radar chart/spider web plot and then classified them based on their distinct combination of knowledge and corresponding health-related behaviors. The clusters were classified based on data interpretation and consensus by the authors^26,27^. The groups were defined relative to each other to establish a suitable reference for comparison and interpretation. Descriptive statistics were used to describe the resulting clusters. Chi-square tests and Analysis of Variance (ANOVA), followed by a Bonferroni corrected Tukey’s Test for post-hoc pairwise comparison, were conducted to determine statistically significant differences between clusters. To better contextualize our results, we compared demographics of our sample with the most recent U.S. census data (2023)^28^.

### Network analysis

To identify the most important facilitator and barrier to practicing healthy behavior, we performed a correlation network analysis^29^. We included knowledge, practice of healthy behaviors, facilitators, and barriers as binary variables and demographics as categorical variables to minimize noise and reduce complexity. We used Cramer’s V to calculate the correlations between these variables, which were visualized in a correlation matrix. Thresholding is widely used in correlation network construction^30,31^. Previous studies often employe thresholds ranging from 0.1 to 0.5, with stepwise increments^31,32^. Following this rationale, we tested different thresholds to identify the most interpretable correlation network (0.1–0.5, in 0.1 increments). We identified facilitators and barriers to knowledge and practice of healthy behavior based on centrality matrices (degree centrality, betweenness centrality, and closeness centrality)^33^. The network graph was visualized using the Fruchterman-Reingold layout, with nodes color-coded by category and edges thickened according to the strength of the correlations^29^.

A background and rationale for both the cluster and network analysis, accompanied by a brief tutorial on how to interpret the subsequent figures, is provided in the supplementary methods.

### Sensitivity analysis

As a sensitivity analysis to confirm the clustering results, we compared the hierarchical clustering results to those obtained from k-means clustering. We calculated the Fowlkes-Mallows Index (FMI)^34^ to assess the similarity between resulting clusters. Additionally, we examined the distributions of knowledge and practice of healthy behaviors across both methods to ascertain cluster similarity.

A two-tailed p-value of 0.05 was considered statistically significant. All analyses were performed in RStudio version 4.4.1. The study was conducted per the STROBE guidelines. All methods were performed in accordance with the relevant guidelines and regulations.

## Results

### Cohort characteristics

One thousand five hundred participants were invited to the survey, of which 1,478 (98.5%) completed it (Figure S1). The baseline characteristics of the cohort are presented in Table 1, and differences with the most recent U.S. Census data are presented in Table S2. The mean age of participants was 45.5 (SD 15.9) years, of which 754 were female (51.8%), and 1106 (74.8%) were non-Hispanic White.

**Table 1:** Baseline characteristics.

Demographics		Included cohort (*N* = 1478)
Age, mean (SD)		45.5 (15.9)
Female, N (%)		754 (51.8)
Race/ethnicity, N (%)	Non-Hispanic White	1106 (75.3)
	Non-Hispanic Black/ African American	192 (13.1)
	Asian	91 (6.2)
	Hispanic/Latino/Spanish	62 (4.2)
	Other	17 (1.2)
Education, N (%)	High school diploma or less	453 (31.0)
	Associates degree	713 (48.8)
	Graduate degree	295 (20.2)
Marital status, N (%)	Never married	611 (42.6)
	Currently married	603 (42.0)
	Separated, divorced, widowed	221 (15.4)
Employment, N (%)	Employed	1008 (69.9)
	Unemployed	156 (10.8)
	Not in labor force	278 (19.3)
Personal income, N (%)	$ 0 - $ 10.275	319 (21.6)
	$ 10.276 - $ 41.775	492 (33.4)
	$41.776 - $89.075	455 (30.9)
	≥$ 89.076	208 (14.1)

Baseline characteristics. N denotes the number of participants. % proportions.

### Knowledge and practice of healthy behaviors

Of 1,478 participants who filled out the survey, 1392 (94%) had complete data on the knowledge and practice of healthy behaviors of the eight included risk factors. The proportions of knowledge and practice of healthy behavior for the complete cohort is presented in Table 2. Knowledge of the importance of individual risk factors ranged from 70.3% (*N* = 978) for having a purpose in life to 95.7% (*N* = 1,332) for a healthy diet. The proportion of participants practicing healthy behaviors was lower than the proportions of knowledge for all factors, ranging from 32.7% (*N* = 455) for regular sleep to 81.9% (*n* = 1,140) for not smoking.

**Table 2:** Knowledge and practice of healthy behavior.

Risk factor	Knowledge that a risk factor is important *N* = (%)	Practicing healthy behavior *N* = (%)
Alcohol use	1183 (85.0%)	1027 (73.8%)
Healthy diet	1332 (95.7%)	575 (41.3%)
Smoking	1307 (93.9%)	1140 (81.9%)
Consistent Physical activity	1314 (94.7%)	508 (36.5%)
Regular Sleep schedule	1326 (95.3%)	455 (32.7%)
Manageable Stress	1318 (94.7%)	631 (45.3%)
Presence of social relationship	990 (71.1%)	889 (63.9%)
Purpose in life	978 (70.3%)	903 (64.9%)

Knowledge and practices of healthy behavior. N denotes the number of participants. % the percentage.

### Cluster analysis

**Fig. 1:**
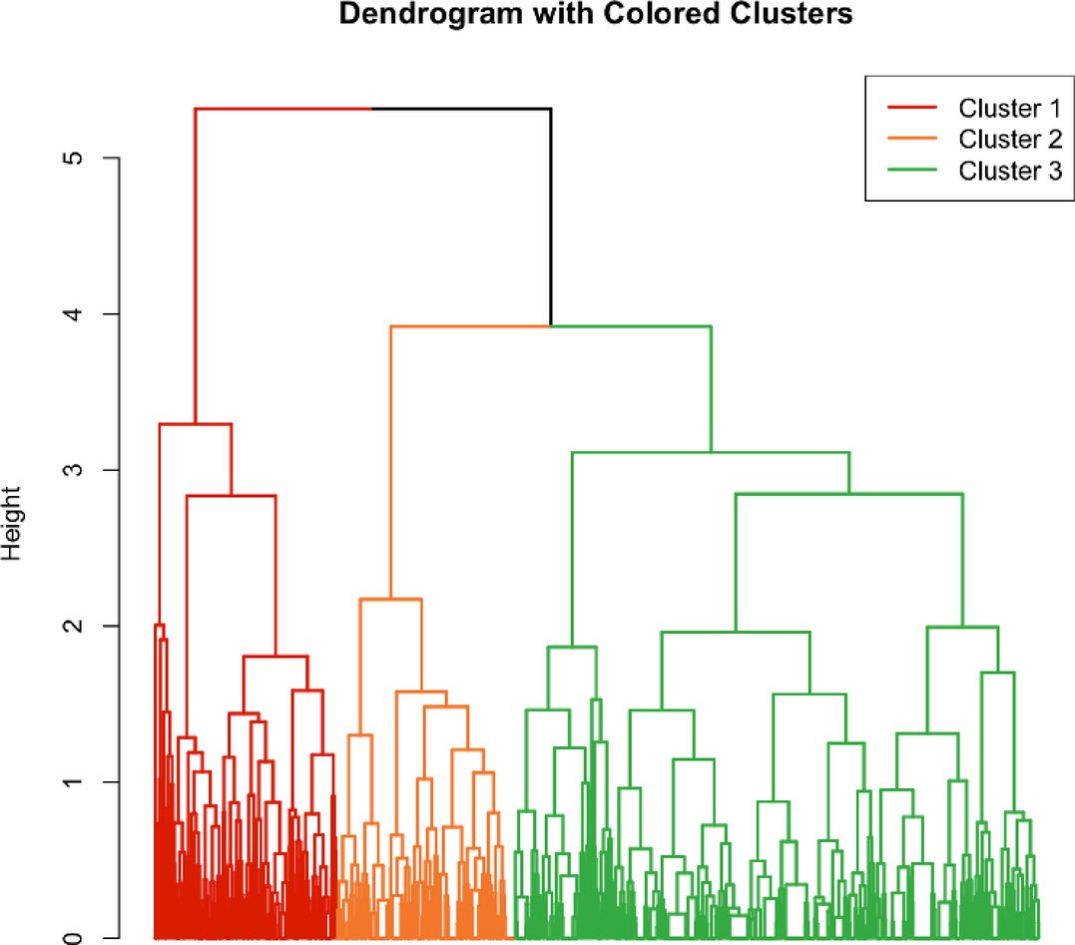
Cluster analysis dendogram. The dendrogram displays the clusters. Each branch corresponds to an individual participant, and branches closer together indicate more similar responses. The height of the branches reflects the dissimilarity between clusters. A horizontal cutoff at a selected height (based on the within-cluster sum of squares) determines the final clusters. Cluster cut-off was at height 4, creating 3 clusters. Cluster 1 (left) is colored red, cluster (2) (middle) is colored orange, and cluster 3 (right) is colored green.

**Fig. 2:**
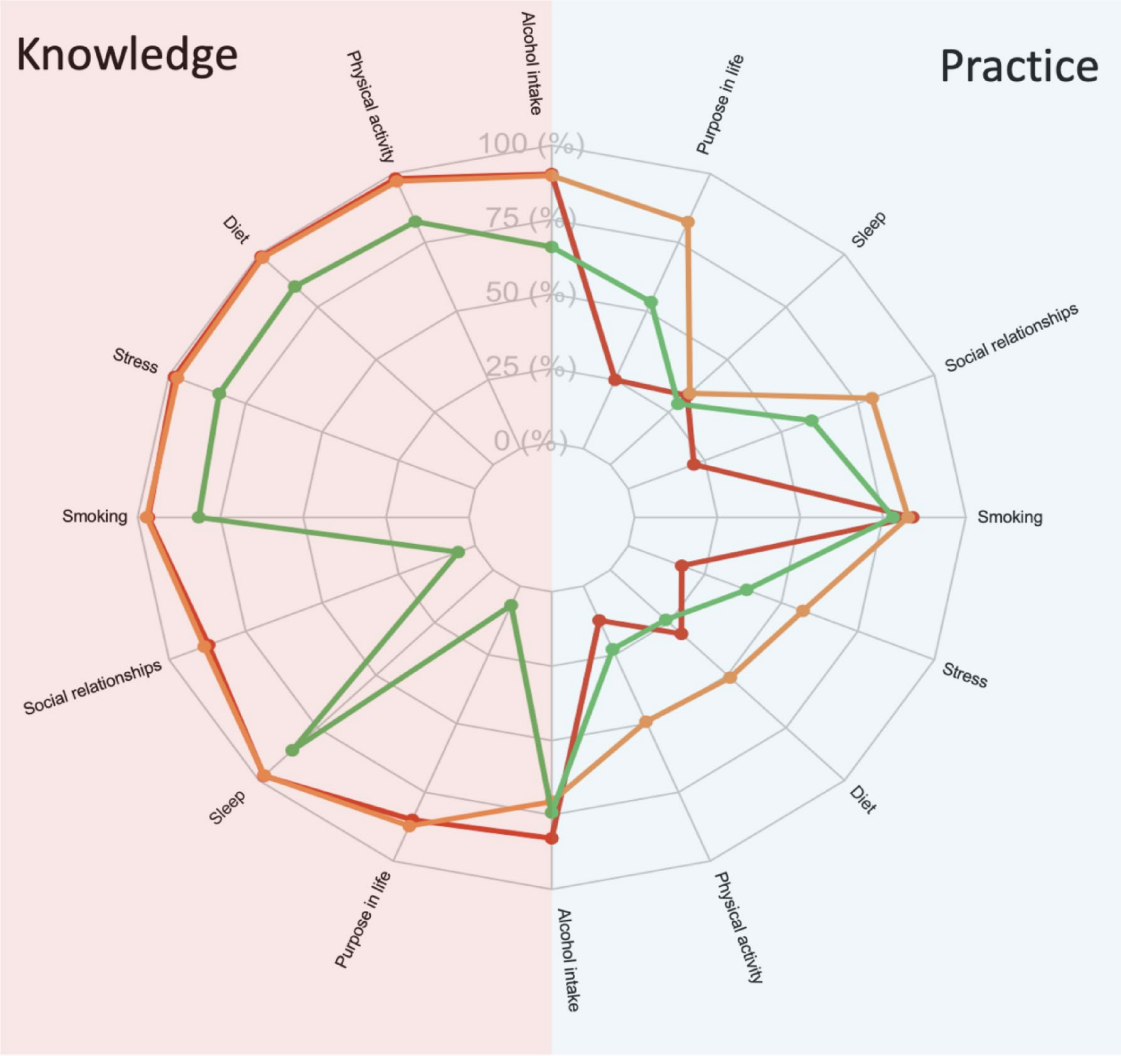
Radar plot. Knowledge and practice of healthy behavior per cluster. Cluster 1 (high knowledge/poor practice) is colored red, cluster 2 (high knowledge/good practice) is colored orange, and cluster 3 (lower knowledge/poor practice) is colored green. The left side (pink background) of the radar plot presents the % of individuals that have the knowledge that a certain risk facor is a useful contributor to maintaining brain health. The right side (blue background) of the plot presents the % of individuals in a cluster that practice a healthy behavior.

Based on the within-cluster sum of squares plot (Figure S2), the optimal number of clusters was determined to be three (Fig. 1), with moderate to high cluster stability (Jaccard bootstrap mean 0.60–0.73) (Table S3). The three identified clusters were defined as cluster I: high level of knowledge and poor practice of healthy behavior, cluster II: high knowledge and good practice, and cluster III: lower knowledge and poor practice (Fig. 2). The distribution of the knowledge and practice of healthy behaviors per cluster is presented in Table S4.

### Comparing the clusters

Overall, the high knowledge and good practice cluster tended to be older, more highly educated, and have higher income levels. They also reported fewer barriers and more facilitators to engaging in healthy behaviors (*p* < 0.05 for all but one comparison; Table 3).

**Table 3:** Characteristics per cluster.

Variable		Cluster 1 High Knowledge Poor Practice *N* = 280	Cluster 2 High Knowledge Good Practice *N* = 825	Cluster 3 Lower Knowledge Poor Practice *N* = 287	*P*- value
**Demographics**					
Age		42.9 (15.0)^2^	47.3 (16.2)^1,3^	42.6 (15.4)^2^	< 0.001
Female		167 (60.7)^2,3^	411 (50.5)^1^	143 (50.0)^1^	0.010
Race/ethnicity	Non-Hispanic White	203 (72.5)	630 (77.0)	206 (72.3)	0.097
	Non-Hispanic Black/African American	36 (12.9)	103 (12.6)	44 (15.4)	
	Asian	22 (7.9)	50 (6.1)	15 (5.3)	
	Hispanic/Latino/Spanish	13 (4.6)	31 (3.8)	13 (4.6)	
	Other	6 (2.1)	4 (0.5)	7 (2.5)	
Education	High school diploma or less	105 (38.0)^2^	208 (25.5)^1,3^	116 (41.0)^2^	< 0.001
	Associates degree	131 (47.5)	430 (52.6)^3^	117 (41.3)^2^	
	Graduate degree	40 (14.5)^2^	179 (21.9)^1^	50 (17.7)	
Marital status	Never married	137 (50.0)^2^	301 (37.7)^1,3^	143 (51.1)^2^	< 0.001
	Currently married	81 (29.6)^2^	380 (47.6)^1,3^	99 (35.4)^2^	
	Separated, divorced, widowed	56 (20.4)	117 (14.7)	38 (13.6)	
Employment	Employed	174 (63.0)^2^	585 (72.6)^1^	187 (67.0)	< 0.001
	Unemployed	46 (16.7)^2^	58 (7.2)^1,3^	42 (15.1)^2^	
	Not in labor force	56 (20.3)	163 (20.2)	50 (17.9)	
Personal income	$ 0 - $ 10.275	77 (27.6) ^2^	145 (17.6)^1,3^	84 (29.4)^2^	< 0.001
	$ 10.276 - $ 41.775	103 (36.9)	271 (32.9)	93 (32.5)	
	$41.776 - $89.075	76 (27.2)	272 (33.0)^3^	72 (25.2)^2^	
	≥$ 89.076	23 (8.2)^2^	138 (16.5)^1^	37 (12.9)	
**Facilitators and/or barriers – Agree with statement N(%)**					
Changing lifestyle and health habits is difficult to maintain over a long period of time		194 (69.3)^2,3^	443 (53.8)^1^	171 (59.8)^1^	< 0.001
I am too busy to change my lifestyle and health habits		59 (21.1)^2^	86 (10.1)^1,3^	50 (17.4)^2^	< 0.001
My financial situation does not allow me to change my lifestyle and behavior		125 (44.8)^2^	143 (17.4)^1,3^	115 (40.1)^2^	< 0.001
Learning more about dementia and stroke motivates me to change my lifestyle		211 (75.6)^2,3^	701 (85.1)^1,3^	185 (64.9)^1,2^	< 0.001
Having risk factors for dementia and stroke makes me want to change my lifestyle		230 (82.1)^3^	710 (86.1)^3^	206 (71.1)^1,2^	< 0.001
I am confident that I can change my lifestyle and behaviour so I can reduce the risk of developing dementia and stroke		161 (57.7)^2^	652 (79.1)^1,3^	151 (52.6)^2^	< 0.001
I feel at high risk of developing dementia		116 (41.4)^2,3^	210 (25.5)^1,3^	93 (32.4)^1,2^	< 0.001
I feel at high risk of having a stroke		99 (35.4)^2^	194 (23.5)^1^	86 (30.1)	< 0.001
I have ever known someone with dementia or stroke		216 (77.1)^2^	690 (83.7)^1,3^	222 (77.4)^2^	0.001
I have ever been a caregiver for someone with dementia or stroke		79 (28.2)	215 (26.1)	64 (22.3)	0.26

Demographics, facilitators and barriers stratified by cluster. N denotes the number of participants. % proportions. Superscript numbers indicate statistically significant differences (p < 0.05) between the corresponding cluster, for example: cluster 2 was significantly older than cluster 1 and 3, while there were no statistical significant differences in age between cluster 1 and 3.

### Demographics

The high knowledge and good practice cluster was older, with a mean age of 47.3 years, compared to the other clusters, which have mean ages ranging from 42.6 to 42.9 years (*p* < 0.001). The high knowledge and good practice cluster also had fewer people in the lowest education category (25.5% vs. 38.0–41.0%, *p* < 0.001), lower unemployment rates (7.2% vs. 15.1–16.7%, *p* < 0.001), and fewer people in the lowest income category (17.6% vs. 27.6–29.4%, *p* < 0.001) as compared to the other clusters. The high knowledge and poor practice cluster had more females (60.7%) than the other clusters (50.0-50.5%, *p* = 0.01).

### Facilitators and barriers

The differences in perceived facilitators and barriers is also presented in Table 3. The high knowledge and good practice cluster had the lowest proportion of individuals who perceive the barriers: ‘*Changing lifestyle and health habits are difficult to maintain over a long period of time’* (53.8% versus [vs] 59.8-69.3%, p < 0.001), ‘*I am too busy to change my lifestyle and health habits*’ (10.1% vs. 17.4%-21.1%, p < 0.001), and ‘*My financial situation does not allow me to change my lifestyle and behavior*’ (17.4% vs. 40.1%-44.8%, p < 0.001). Additionally, the high knowledge and good practice cluster the highest proportion of individuals who perceive the facilitators: ‘*Learning more about dementia and stroke motivates me to change my lifestyle*’ (85.1% vs. 64.9%-75.6%, p < 0.001), ‘*Having risk factors for dementia and stroke makes me want to change my lifestyle*’ (86.1% vs. 71.1%-82.1%, p < 0.001), and ‘*I am confident that I can change my lifestyle and behavior so I can reduce the risk of developing dementia and stroke*’ (79.1% vs. 52.6%-57.7%, p < 0.001). Furthermore, this high knowledge and good practice cluster had the highest proportion of people who ever knew someone with dementia or stroke (83.7% vs. 77.1%-77.4%, p < 0.001). The high knowledge and poor practice and the lower knowledge and poor practice clusters had higher proportions of individuals who feel at risk of developing dementia (41.4% and 32,4, respectively vs. 25.5%, p = 0.001) and feel at high risk of developing stroke (35.4% vs. 23.5%, p < 0.001).

### Network analysis

**Fig. 3:**
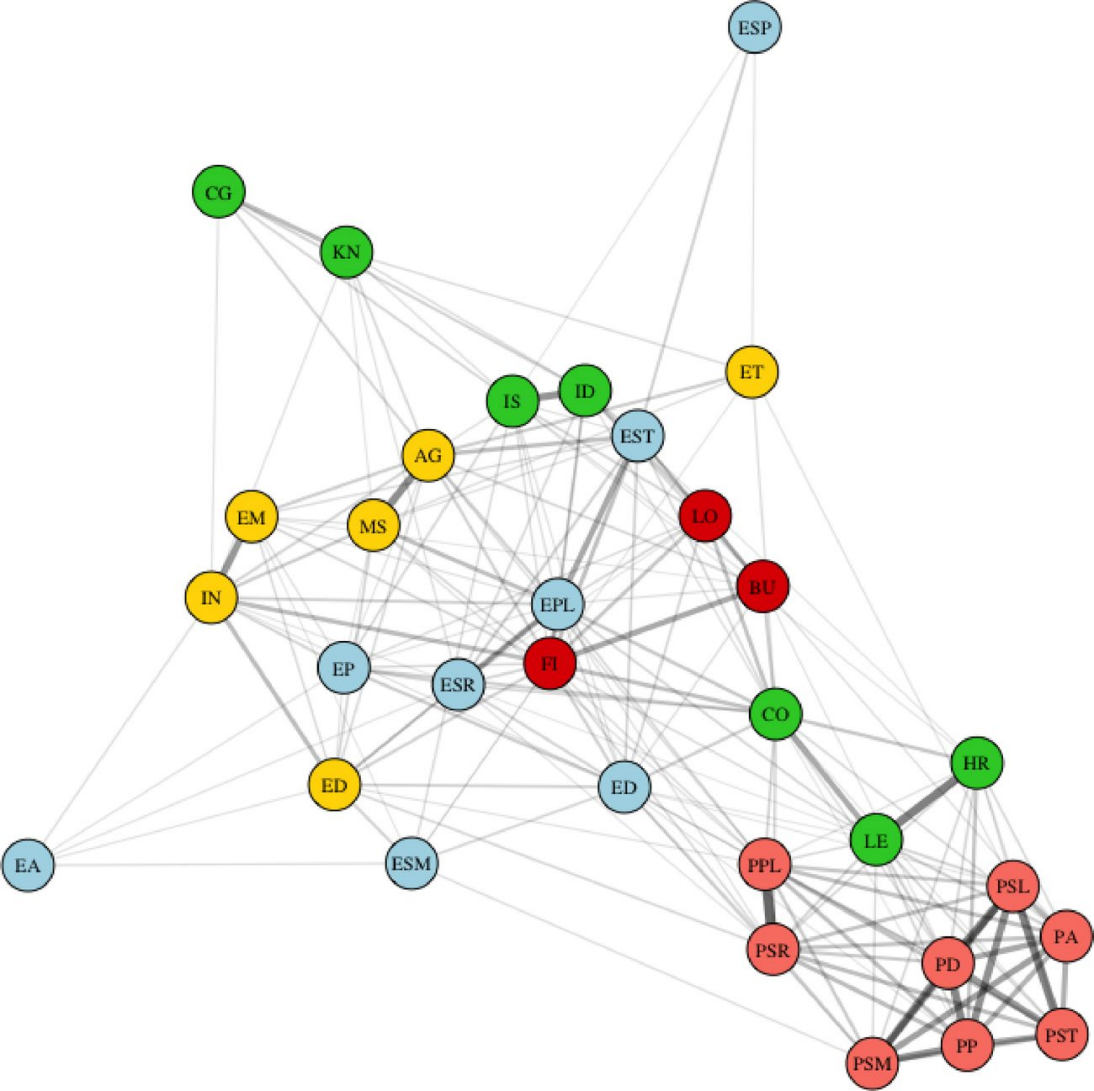
Network analysis. Correlation Network with nodes color-coded based on their categories and edges thickened according to the strength of the correlations.work analysis. Knowledge (Pink Nodes): PA: Knowing that low alcohol is a contributor to keeping your brain healthy, P.D.: Knowing that a healthy diet is a contributor to keeping your brain healthy, P.P.: Knowing that physical activity is a contributor to keeping your brain healthy, PPL: Knowing that purpose in life is a contributor to keeping your brain healthy, PSL: Knowing that regular sleep is a contributor to keeping your brain healthy, PSM: Knowing that not smoking is a contributor to keeping your brain healthy, PSR: Knowing that social relations are a contributor to keeping your brain healthy, PST: Knowning that manageable levels of stress are a contributor to keeping your brain healthy. Health Related Behaviors (Blue Nodes): E.A.: Practice of healthy alcohol behavior, E.D.: Practice of healthy diet behavior, EPL: Having purpose in life, E.P.: Practice of healthy physical activity behavior., ESP: Practice of healthy sleep behavior, ESM: Not smoking, ESR: Having at least two people, other than partner or children, to talk to, EST: Having manageable levels of stress. Demographics (Yellow Nodes):, AG: Age, E.T.: Ethnicity, E.D.: Education, MS: Marital status, EM: Employment, IN: Income. Facilitators (Green Nodes): L.E.: Learning more about dementia and stroke motivates me to change my lifestyle, I.S.: I feel at high risk of having a stroke. H.R.: Having risk factors for dementia and stroke makes me want to change my lifestyle, CO: I am confident that I can change my lifestyle and behavior so I can reduce the risk of developing dementia and stroke, ID: I feel at high risk of developing dementia, L.O.: Changing lifestyle and health habits is difficult to maintain over a long period of time, K.N.: I have ever known someone with dementia or stroke, C.G.: I have ever been a caregiver for someone with dementia or stroke. Barriers (Red Nodes): F.I.: My financial situation does not allow me to change my lifestyle and behavior, L.O.: Changing lifestyle and health habits is difficult to maintain over a long period of time, B.U.: I am too busy to change my lifestyle and health habits.

Based on the Cramer’s V correlation matrix (Figure S3), a correlation network analysis was plotted (Fig. 3) with a correlation threshold of 0.1. An overview of the centrality matrices for all included variables is presented in Figure S4. The barrier ‘*my financial situation does not allow me to change my lifestyle and behavior*’ and the facilitator ‘*I feel at high risk of having a stroke’* had the highest centrality matrices and were both correlated with the practice of healthy behavior.

### Sensitivity analysis

A sensitivity analysis utilizing a k-means clustering method identified similar behavioral profiles as the hierarchial clustering; cluster I: high knowledge and poor practice, cluster II: high knowledge and good practice, and cluster III: lower knowledge and poor practice (Table S5, Figure S5). The Fowlkes-Mallows Index (FMI) comparing the hierarchical and k-means clustering was 0.60.

### Post-hoc power calculation

We conducted a post-hoc power analysis to determine the minimum effect size our study was powered to detect in a chi-squared test comparing proportions across three clusters (*n* = 280, 825, and 287). Given an alpha of 0.05 and a target power of 80%, our sample was sufficient to detect a small effect size of (Cohen’s w = 0.07), corresponding to a difference in proportions of approximately 3 to 4% points^35^.

## Discussion

In this study of 1,478 U.S. participants, we found that most people had knowledge on the importance of healthy behavior, however fewer people also practiced the corresponding healthy behavior across all eight risk factors. We identified three clusters and found that those in the clusters with lower levels of knowledge and poor practices of healthy behavior were younger, less educated, had lower incomes, perceived more barriers (including financial and time limitations), and fewer facilitators (including levels of motivation and knowing someone with dementia or stroke).

We observed statistically significant inequities in demographic variables across the clusters. We found that socioeconomic variables including income and education were less favorable among the clusters with lower levels of knowledge and lower practice of healthy behavior. Interestingly, these participants were also more likely to perceive barriers and less likely to perceive facilitators towards healthy behavior. This aligns with previous literature, reinforcing the well-documented association between demographic inequities and the ability to adopt preventive health behaviors^36,37^.The discrepancy between knowledge and practice of healthy behavior aligns with previous literature^9,14^, suggesting that while understanding the importance of these risk factors is essential for behavioral change, it is insufficient on its own^8^. These findings align with guidelines from the American Heart Association (AHA) and the American Academy of Neurology (AAN), which advocate for comprehensive prevention strategies that go beyond raising awareness. They underscore the importance of creating opportunities for actual behavioral change to reduce the burden of dementia and stroke^38,39^. We found significant differences in facilitators and barriers to achieving behavioral change among different clusters, aligning with previous findings that show heterogeneity in perceived facilitators and barriers across populations^11,12,40^. The findings that those with lower knowledge and healthy behavior were younger, less educated, had lower incomes, and perceived more barriers and fewer facilitators are consistent with previous literature^10–12^. This could indicate the need for tailored interventions that consider specific demographic and socioeconomic contexts to effectively promote healthy behavior changes^14^.

Our network analysis identified the barrier perceived financial situation and the facilitator feeling at high risk of stroke as the most important within the network. We hypothesize that targeting these factors could be key for future interventions. Previous studies describe the associations between financial barriers and health status, healthcare utilization, incidence, and functional outcome of age-related brain disease^41,42^. However, the optimal way to achieve behavioral change through overcoming financial barriers is uncertain. For example, financial incentives show contradicting results to improvement of sustained healthy behavior changes^43^, while financial barriers on a structural level (e.g., taxes on sugar-sweetened beverages) might be effective^34^. Regarding feeling at high risk of developing a stroke, previous literature has shown that fear of disease could either lead to defensive reactions, such as denial or supression of behavior change or proactive change to mitigate the feared risk^44^.

Some limitations should be considered when interpreting these results. Firstly, due to the study’s cross-sectional design, we cannot determine whether knowledge precedes the practice of healthy behaviors. Consequently, no causal inferences can be made^45^. Second, only one metric, the BCS, was used to define healthy behavior. Other frameworks to define healthy behavior might utilise different cut-off values or include other factors. Nonetheless, the BCS is a robust tool that integrates all eight risk factors from the American Heart Association’s Life’s Essential Eight^5,46^, along with additional social-emotional components^4,21^. Moreover, the BCS has shown a strong association with the incidence of age-related brain diseases, while each of these risk factors has well-documented epidemiological associations with these diseases^47–49^. Third, the correlation threshold for the network analysis was low, with higher thresholds showing no interpretable network. This low level of correlation might be due to the inherent variability and complexity of behavioral science data^35^, which often includes subjective self-reports and can be influenced by a wide range of external factors. Fourth, relying on self-reported data for knowledge and healthy behavior may introduce bias, such as social desirability bias, recall bias, or inaccurate reporting^50^. These biases might have resulted in overreporting healthy practices and underreporting unhealthy behaviors. Fifth, there is an imbalance between the number of assessed barriers and facilitators, with only three barriers compared to seven facilitators. Additionally, participants were not able to identify additional barriers, which may limit the ecological validity and comprehensiveness of our findings. Sixth, clusters were formed in part based on perceived knowledge of risk factors, though differences in this variable were marginal, with agreement rates ranging between 70% and 96%. Lastly, this study consisted of a cohort of U.S. adults who were able to complete an online questionnaire and received compensation for it, which might have further biased our sample and, therefore, affected our generalizability to the general U.S. population. When comparing our baseline demographics with the available U.S. Census data, our sample includes a higher proportion of White individuals, higher proportion of those with a graduate degree, and more individuals in the labor force, yet differences were marginal. These differences may further limit the generalizability of our findings beyond this cohort and could have influenced results.

Despite these notable limitations, our study has several strengths. It includes a large sample of nearly 1,500 U.S. adults, providing a comprehensive overview of knowledge and healthy behaviors. We identified three distinct and easily interpretable clusters, revealing significant differences in demographics, facilitators, and barriers. The sensitivity analysis demonstrated consistent findings across both hierarchical and k-means clustering methods, increasing the reliability of our identified clusters.

Prior research showed that knowledge on the modifiable risk factors of dementia and stroke does often not correlate strongly with corresponding health-related behaviors^10,11,13,14^. This study assessed these relationships in a cohort of the U.S. population and adequately identified subgroups, with an extensive investigation into proposed barriers and facilitators. These insights may allow for tailored U.S. preventive strategies aimed at reducing risks of dementia and stroke. More specifically, our findings may inform how public health efforts promote brain-healthy behaviors by allowing for tailored approaches, taking into consideration the proposed barriers and facilitators. For example: individuals with lower knowledge levels, public health campaigns through mass media (TV, billboards, social media, etc.) have been demonstrated to be effective^51^. Those who have adequate knowledge, but lower practice of healthy behaviors may particularly benefit from targeted interventions explicitly addressing barriers such as time constraints or financial limitations. Behavior-change strategies delivered through primary care settings, especially for high-risk subgroups^52^, or through digital health tools may effectively bridge this gap. Prior literature shows that digital health interventions offer accessible, low-cost solutions by incorporating components such as goal setting, health coaching, self-monitoring, and tailored feedback, which can help to overcome the identified barriers, and have shown promise in reducing dementia and stroke risk^53–55^.

Future directions should focus on developing strategies to improve brain health, considering the differences in knowledge and practices of healthy behavior, barriers and facilitators addressed here. Aligning with previous literature, there is not a one-size-fits-all approach for preventing age-related brain disease^5,56^. Our identification of perceived financial situation as a barrier to lifestyle changes warrants further investigation. Quantitative and qualitative studies are essential to better understand individuals’ specific financial challenges and preferences, which will be crucial for developing accessible, low-cost strategies to improve brain health^38,39^. Moreover, given that feeling at high risk of stroke can either spur proactive behavior or lead to defensive reactions such as denial, future interventions should carefully balance these elements to enhance their effectiveness.

In this study, we found that while most participants knew the importance of modifiable risk factors for brain health, fewer practiced the corresponding healthy behaviors. The different clusters showed significant differences in demographics, barriers, and facilitators. These findings suggest targeted interventions to enhance knowledge in some groups and support the translation of knowledge into practice in others. Future studies should explore how identified key barriers and facilitators can be leveraged to effective preventative strategies.

## Electronic supplementary material

Below is the link to the electronic supplementary material.

Supplementary Material 1

## Data Availability

The datasets generated and/or analysed during the current study are not publicly available due to local data sharing agreement regulations but are available from the corresponding author on reasonable request.

## References

[1] Global Burden of Disease Collaborative Network. *Global Burden of Disease Study 2021 (GBD 2021)* (Institute for Health Metrics and Evaluation (IHME), 2024).

[2] Tsao, C. W. et al. Heart disease and stroke Statistics-2022 update: A report from the American heart association. *Circulation* **145** (8), e153–e639. 10.1161/CIR.0000000000001052 (2022). 35078371 10.1161/CIR.0000000000001052

[3] Livingston, G. et al. Dementia prevention, intervention, and care: 2024 report of the lancet standing commission. *Lancet*10.1016/S0140-6736(24)01296-0 10.1016/S0140-6736(24)01296-0 39096926

[4] Senff, J. et al. Modifiable risk factors for stroke, dementia, and Late-Life depression: A systematic review and DALY weighted risk factors for a composite outcome. *J. Neurol. Neurosurg. Psychiatry*. **0**, 1–13. 10.1136/Jnnp-2024-334925 (2025). 10.1136/jnnp-2024-334925 40180437

[5] Singh, S. D. et al. Brain health begins with brain care. *Lancet Neurol.* **21** (11), 961–962. 10.1016/S1474-4422(22)00397-0 (2022). 36270304 10.1016/S1474-4422(22)00397-0

[6] Biswas, T. et al. Prevalence of multiple non-communicable diseases risk factors among adolescents in 140 countries: A population-based study. *eClinicalMedicine* **52**10.1016/j.eclinm.2022.101591 (2022). 10.1016/j.eclinm.2022.101591 PMC9396043 36016694

[7] Aggarwal, R. et al. Racial/Ethnic disparities in hypertension prevalence, awareness, treatment, and control in the united States, 2013 to 2018. *Hypertension* **78** (6), 1719–1726. 10.1161/HYPERTENSIONAHA.121.17570 (2021). 34365809 10.1161/HYPERTENSIONAHA.121.17570 PMC10861176

[8] Michie, S., van Stralen, M. M. & West, R. The behaviour change wheel: A new method for characterising and designing behaviour change interventions. *Implement. Sci.* **6** (1), 42. 10.1186/1748-5908-6-42 (2011). 21513547 10.1186/1748-5908-6-42 PMC3096582

[9] Budin-Ljøsne, I. et al. Public perceptions of brain health: an international, online cross-sectional survey. *BMJ Open.* **12** (4), e057999. 10.1136/bmjopen-2021-057999 (2022). 35437254 10.1136/bmjopen-2021-057999 PMC9016409

[10] Heger, I. et al. Dementia awareness and risk perception in middle-aged and older individuals: baseline results of the MijnBreincoach survey on the association between lifestyle and brain health. *BMC Public. Health*. **19** (1), 678. 10.1186/s12889-019-7010-z (2019). 31159779 10.1186/s12889-019-7010-z PMC6545627

[11] Glynn, R. W., Shelley, E. & Lawlor, B. A. Public knowledge and Understanding of dementia—evidence from a National survey in Ireland. *Age Ageing*. **46** (5), 865–869. 10.1093/ageing/afx082 (2017). 28531240 10.1093/ageing/afx082

[12] Dukelow, T. et al. Modifiable risk factors for dementia, and awareness of brain health behaviors: results from the five lives brain health Ireland survey (FLBHIS). *Front. Psychol.* **13**10.3389/fpsyg.2022.1070259 (2023). https://www.frontiersin.org/articles/10.3389/fpsyg.2022.1070259 PMC9879702 36710802

[13] Kulmala, J. et al. Facilitators and barriers to implementing lifestyle intervention programme to prevent cognitive decline. *Eur. J. Pub. Health*. **31** (4), 816–822. 10.1093/eurpub/ckab087 (2021). 34448856 10.1093/eurpub/ckab087 PMC8505000

[14] Kelly, M. P. & Barker, M. Why is changing health-related behaviour so difficult? *Public. Health*. **136**, 109–116. 10.1016/j.puhe.2016.03.030 (2016). 27184821 10.1016/j.puhe.2016.03.030 PMC4931896

[15] Holder, C., Krishnamurthi, R. & Theadom, A. Exploring facilitators and barriers to long-term behavior change following health–wellness coaching for stroke prevention: A qualitative study conducted in Auckland, new Zealand. *Brain Behav.* **13** (1), e2671. 10.1002/brb3.2671 (2023). 36510702 10.1002/brb3.2671 PMC9847597

[16] Prolific Published online 2014. https://www.prolific.com

[17] United States Census Bureau. The American Community Survey Questionnaire. (2020).

[18] Grech, R. & Grech, P. The stroke knowledge assessment tool (SKAT): development, reliability and validity. *J. Med. Health Stud.* **2**, 81–88 (2021).

[19] Annear, M. J. et al. Dementia knowledge assessment scale (DKAS): confirmatory factor analysis and comparative subscale scores among an international cohort. *BMC Geriatr.* **17** (1), 168. 10.1186/s12877-017-0552-y (2017). 28760154 10.1186/s12877-017-0552-y PMC5537989

[20] Kim, S., Sargent-Cox, K., Cherbuin, N. & Anstey, K. J. Development of the motivation to change lifestyle and health behaviours for dementia risk reduction scale. *Dement. Geriatr. Cogn. Dis. Extra*. **4** (2), 172–183. 10.1159/000362228 (2014). 25028583 10.1159/000362228 PMC4086035

[21] Singh, S. et al. The Predictive Validity of A Brain Care Score for Dementia and Stroke: data from the UK Biobank cohort. *Front Neurol*. Volume 14-2023 |. doi:10.3389/fneur.2023.1291020 10.3389/fneur.2023.1291020 PMC10725202 38107629

[22] Rivier, C. A. et al. Brain care score and neuroimaging markers of brain health in asymptomatic Middle-Age persons. *Neurology* **103** (4), e209687. 10.1212/WNL.0000000000209687 (2024). 39052961 10.1212/WNL.0000000000209687 PMC11760050

[23] Singh, S. D. et al. The predictive validity of a brain care score for late-life depression and a composite outcome of dementia, stroke, and late-life depression: data from the UK biobank cohort. *Front. Psychiatry*. **15**10.3389/fpsyt.2024.1373797 (2024). 10.3389/fpsyt.2024.1373797 PMC11301016 39109366

[24] Rodriguez, M. Z. et al. Clustering algorithms: A comparative approach. *PLoS One*. **14** (1), e0210236. 10.1371/journal.pone.0210236 (2019). 30645617 10.1371/journal.pone.0210236 PMC6333366

[25] Rousseeuw, P. J. Silhouettes. A graphical aid to the interpretation and validation of cluster analysis. *J. Comput. Appl. Math.* **20**, 53–65 (1987).

[26] Frades, I. & Matthiesen, R. Overview on techniques in cluster analysis. Bioinformatics Methods in Clinical Research. Vol 593. Methods in Molecular Biology. Humana; :81–107. doi:10.1007/978-1-60327-194-3_5 (2010). 10.1007/978-1-60327-194-3_5 19957146

[27] Zhang, R. et al. Associations of dietary patterns with brain health from behavioral, neuroimaging, biochemical and genetic analyses. *Nat. Mental Health*. **2** (5), 535–552. 10.1038/s44220-024-00226-0 (2024).

[28] United States Census Bureau. Census Results. Accessed March 18, 2025. (2025). https://www.census.gov/data.html

[29] Costantini, G. et al. State of the aRt personality research: A tutorial on network analysis of personality data in R. *J. Res. Pers.* **54**, 13–29. 10.1016/j.jrp.2014.07.003 (2015).

[30] Masuda, N., Boyd, Z. M., Garlaschelli, D. & Mucha, P. J. Correlation networks: interdisciplinary approaches beyond thresholding. *Published Online*. 10.48550/ARXIV.2311.09536 (2023). 10.1016/j.physrep.2025.06.002 PMC12345407 40814354

[31] Garrison, K. A., Scheinost, D., Finn, E. S., Shen, X. & Constable, R. T. The (in)stability of functional brain network measures across thresholds. *NeuroImage* **118**, 651–661. 10.1016/j.neuroimage.2015.05.046 (2015). 26021218 10.1016/j.neuroimage.2015.05.046 PMC4554838

[32] Buckner, R. L. et al. Cortical hubs revealed by intrinsic functional connectivity: mapping, assessment of stability, and relation to Alzheimer’s disease. *J. Neurosci.* **29** (6), 1860–1873. 10.1523/JNEUROSCI.5062-08.2009 (2009). 19211893 10.1523/JNEUROSCI.5062-08.2009 PMC2750039

[33] Rodebaugh, T. L. et al. Does centrality in a cross-sectional network suggest intervention targets for social anxiety disorder? *J. Consult Clin. Psychol.* **86** (10), 831–844. 10.1037/ccp0000336 (2018). 30265042 10.1037/ccp0000336 PMC6166439

[34] Rachwał, A. et al. Determining the quality of a dataset in clustering terms. *Appl. Sci.* **13** (5). 10.3390/app13052942 (2023).

[35] Cohen, J. Statistical power analysis for the behavioral sciences. *Routledge*10.4324/9780203771587 (1988). 2nd Ed.

[36] Reshetnyak, E. et al. Impact of multiple social determinants of health on incident stroke. *Stroke* **51** (8), 2445–2453. 10.1161/STROKEAHA.120.028530 (2020). 32673521 10.1161/STROKEAHA.120.028530 PMC9264323

[37] Short, S. E. & Mollborn, S. Social determinants and health behaviors: conceptual frames and empirical advances. *Curr. Opin. Psychol.* **5**, 78–84. 10.1016/j.copsyc.2015.05.002 (2015). 26213711 10.1016/j.copsyc.2015.05.002 PMC4511598

[38] Lazar, R. M. et al. A primary care agenda for brain health: A scientific statement from the American heart association. *Stroke* **52** (6), e295–e308. 10.1161/STR.0000000000000367 (2021). 33719523 10.1161/STR.0000000000000367 PMC8995075

[39] Rost, N. S. et al. The brain health imperative in the 21st Century—A call to action. *Neurology* **101** (13), 570–579. 10.1212/WNL.0000000000207739 (2023). 37730439 10.1212/WNL.0000000000207739 PMC10558159

[40] Müller-Nordhorn, J. et al. Knowledge about risk factors for stroke. *Stroke* **37** (4), 946–950. 10.1161/01.STR.0000209332.96513.82 (2006). 16514090 10.1161/01.STR.0000209332.96513.82

[41] Addo, J. et al. Socioeconomic status and stroke. *Stroke* **43** (4), 1186–1191. 10.1161/STROKEAHA.111.639732 (2012). 22363052 10.1161/STROKEAHA.111.639732

[42] Lu, K., Xiong, X., Horras, A., Jiang, B. & Li, M. Impact of financial barriers on health status, healthcare utilisation and economic burden among individuals with cognitive impairment: a National cross-sectional survey. *BMJ Open.* **12** (5), e056466. 10.1136/bmjopen-2021-056466 (2022). 35508339 10.1136/bmjopen-2021-056466 PMC9073389

[43] Vlaev, I., King, D., Darzi, A. & Dolan, P. Changing health behaviors using financial incentives: a review from behavioral economics. *BMC Public. Health*. **19** (1), 1059. 10.1186/s12889-019-7407-8 (2019). 31391010 10.1186/s12889-019-7407-8 PMC6686221

[44] Moussaoui, L. S., Claxton, N. & Desrichard, O. Fear appeals to promote better health behaviors: an investigation of potential mediators. *Health Psychol. Behav. Med.* **9** (1), 600–618. 10.1080/21642850.2021.1947290 (2021). 34285825 10.1080/21642850.2021.1947290 PMC8266257

[45] Fedak, K. M., Bernal, A., Capshaw, Z. A. & Gross, S. Applying the Bradford hill criteria in the 21st century: how data integration has changed causal inference in molecular epidemiology. *Emerg. Themes Epidemiol.* **12**, 14. 10.1186/s12982-015-0037-4 (2015). 26425136 10.1186/s12982-015-0037-4 PMC4589117

[46] Lloyd-Jones, D. M. et al. Life’s essential 8: updating and enhancing the American heart association’s construct of cardiovascular health: A presidential advisory from the American heart association. *Circulation* **146** (5). 10.1161/CIR.0000000000001078 (2022). 10.1161/CIR.0000000000001078 PMC10503546 35766027

[47] Elovainio, M. et al. Association of social isolation, loneliness and genetic risk with incidence of dementia: UK biobank cohort study. *BMJ Open.* **12** (2). 10.1136/bmjopen-2021-053936 (2022). 10.1136/bmjopen-2021-053936 PMC8867309 35197341

[48] Sutin, D. A. R. et al. Sense of meaning and purpose in life and risk of incident dementia: new data and meta-analysis. *Arch. Gerontol. Geriatr.* **105**, 104847. 10.1016/j.archger.2022.104847 (2023). 36347158 10.1016/j.archger.2022.104847 PMC10015423

[49] Booth, J. et al. Evidence of perceived psychosocial stress as a risk factor for stroke in adults: A meta-analysis. *BMC Neurol.* **15** (1). 10.1186/s12883-015-0456-4 (2015). 10.1186/s12883-015-0456-4 PMC4643520 26563170

[50] Rosenman, R., Tennekoon, V. & Hill, L. G. Measuring bias in self-reported data. *Int. J. Behav. Healthc. Res.* **2** (4), 320–332. 10.1504/IJBHR.2011.043414 (2011). 25383095 10.1504/IJBHR.2011.043414 PMC4224297

[51] Wakefield, M. A., Loken, B. & Hornik, R. C. Use of mass media campaigns to change health behaviour. *Lancet* **376** (9748), 1261–1271. 10.1016/S0140-6736(10)60809-4 (2010). 20933263 10.1016/S0140-6736(10)60809-4 PMC4248563

[52] Hulscher, M. E., Wensing, M., van Der Weijden, T. & Grol, R. Interventions to implement prevention in primary care. *Cochrane Database Syst. Rev.* (1), CD000362. 10.1002/14651858.CD000362 (2001). 10.1002/14651858.CD000362 11279688

[53] Liu, S. et al. Mobile health as a viable strategy to enhance stroke risk factor control: A systematic review and meta-analysis. *J. Neurol. Sci.* **378**, 140–145. 10.1016/j.jns.2017.04.050 (2017). 28566151 10.1016/j.jns.2017.04.050 PMC5503473

[54] van Moll, E. P. et al. Prevention of dementia using mobile phone applications (PRODEMOS): a multinational, randomised, controlled effectiveness-implementation trial. *Lancet Healthy Longev.* **5** (6), e431–e442. 10.1016/S2666-7568(24)00068-0 (2024). 38763155 10.1016/S2666-7568(24)00068-0

[55] Jakob, R. et al. Factors influencing adherence to mHealth apps for prevention or management of noncommunicable diseases: systematic review. *J. Med. Internet Res.* **24** (5), e35371. 10.2196/35371 (2022). 35612886 10.2196/35371 PMC9178451

[56] World Health Organization. *A Guide To Implementation Research in the Prevention and Control of Noncommunicable Diseases* (World Health Organization, 2016). https://iris.who.int/handle/10665/252626

