# Supplementary material for: Knowledge and practice of healthy behaviors for dementia and stroke prevention in a United States cohort
Source: Sci Rep. 2025 Apr 30;15:15172. doi: 10.1038/s41598-025-99246-8 (PMC12044067; doi:10.1038/s41598-025-99246-8)
Supplement: Supplementary file 1 — Supplementary Material 1 [file 41598_2025_99246_MOESM1_ESM.docx]

**Knowledge and Practice of Healthy Behaviors for Dementia and Stroke Prevention in a United States Cohort**

Jasper R. Senff, MD^1-5*^; Reinier W.P. Tack, MD^1-5^; Benjamin Y.Q. Tan, MD^1-4,6^; Savvina Prapiadou MD^1-4^; Tamara N. Kimball MD^1-4,7^; Sharon Ng BSc^1-4^; Jonathan Duskin MD^1-4^; Mark Jun Shah-Ostrowski, MD^8^; Courtney Nunley^1^; H. Bart Brouwers MD PhD^5^; Zeina Chemali MD MPH^1,2,9^; Gregory Fricchione MD, PhD^1,10^; Rudolph E. Tanzi PhD^1-4^; Koen Pouwels PhD^8^; Jonathan Rosand, MD MSc^1-4^; Nirupama Yechoor MD^1-4^; Christopher D. Anderson MD MMsc^1-4,7^; Sanjula D. Singh, MD PhD MSc^1-5^

**Affiliations:**

1. Henry and Allison McCance Center for Brain Health, Massachusetts General Hospital, Boston, MA, USA
2. Department of Neurology, Massachusetts General Hospital, Boston, MA, USA
3. Broad Institute of MIT and Harvard, Cambridge, MA, USA
4. Center for Genomic Medicine, Massachusetts General Hospital, Boston, MA, USA
5. Department of Neurology and Neurosurgery, Brain Center Rudolf Magnus, University Medical Center Utrecht, Utrecht, The Netherlands
6. Division of Neurology, Department of Medicine, National University Hospital, Singapore
7. Department of Neurology, Brigham and Women’s Hospital, Boston, MA, USA
8. Nuffield Department of Population Health, University of Oxford, Oxford, United Kingdom
9. Division of Neuropsychiatry, Massachusetts General Hospital, Boston, MA, United States
10. Benson-Henry Institute for Mind Body Medicine, Massachusetts General Hospital, Boston, MA, United States

**Corresponding author:**

- Jasper R. Senff MD
- Email address: jasper_senff@hotmail.com
- Address: McCance Center for Brain Health, Massachusetts General Hospital, Harvard Medical School, Revolution Drive 399, Sommerville, 02145 MA, United States of America

Table of Contents

[Supplementary Methods 3](#_Toc194047294)

[Tables 5](#_Toc194047295)

[Table S1: HOBCAS Questionnaire 5](#_Toc194047296)

[Table S2: Comparing Cohort to U.S. Census data. 11](#_Toc194047297)

[Table S3: Jaccard bootstrap 12](#_Toc194047298)

[Table S4: Distribution of knowledge and practice of healthy behavior 13](#_Toc194047299)

[Table S5: Sensitivity analysis: distribution of hierarchical clustering and k means clustering. 14](#_Toc194047300)

[Figures 15](#_Toc194047301)

[Figure S1: Flowchart 15](#_Toc194047302)

[Figure S2: total-within cluster sum of squares 16](#_Toc194047303)

[Figure S3: correlation matrix 17](#_Toc194047304)

[Figure S4: Centrality plots 18](#_Toc194047305)

[Figure S5: Sensitivity analysis – differences between hierarchical and k-means cluster 19](#_Toc194047306)

# Supplementary Methods

**Cluster analysis**

**Background and rationale for a cluster analysis**

Hierarchical clustering is an unsupervised learning method that groups participants based on multiple variables without predefined classifications^1^. This method is particularly suitable for analyzing complex, high-dimensional categorical data—such as human behavioral characteristics—because it identifies naturally occurring groupings that may not be readily apparent through standard descriptive approaches^2^. We performed hierarchical cluster methods rather than descriptive analysis to reduce dimensionality of the included data and identify natural patterns of knowledge and practice, while retaining interpretable results. We opted for hierarchical clustering instead of descriptive categorization to effectively reduce data dimensionality, detect natural patterns of knowledge and behavior, and preserve interpretability. Importantly, by avoiding arbitrary cut-offs (e.g., labeling groups based on a fixed threshold) and refraining from comparing all variables with each other, we aimed to minimize potential biases arising from subjective decisions and decrease the likelihood of type I errors due to multiple testing^3,4^.

**Interpretation of the cluster analysis figures**

*Figure 1. Cluster Analysis Dendrogram*

This dendrogram represents the hierarchical clustering of participants based on their knowledge and practice of healthy behaviors. Each branch corresponds to an individual participant, and branches that are closer together indicate individuals with more similar responses^5^. The height of the branches reflects the dissimilarity between clusters—the greater the height, the more distinct the groups. A horizontal cutoff at a selected height (based on within-cluster sum of squares) determined the final three clusters: Cluster 1 (Red): High knowledge, poor practice, Cluster 2 (Orange): High knowledge, good practice, Cluster 3 (Green): Lower knowledge, poor practice.

*Figure 2. Radar plot.*

The radar plot provides a visual comparison of how well each cluster understands and practices different health behaviors^6^. The left side (pink background) represents the percentage of participants in each cluster who recognize a factor as important for brain health (knowledge). The right side (blue background) represents the percentage of participants who actually engage in the corresponding healthy behavior (practice). The colors represent the three identified clusters: Red (Cluster 1): High knowledge, poor practice, Orange (Cluster 2): High knowledge, good practice, Green (Cluster 3): Lower knowledge, poor practice.

**Network analysis**

**Background and rationale for a network analysis**

The network analysis was conducted as an exploratory approach to move beyond descriptive comparisons and examine the structural relationships between knowledge, practice, facilitators, barriers, and demographics. Traditional statistical models often assume that variables operate independently, but health behaviors are shaped by complex, interdependent relationships rather than isolated factors^7^. We performed the network analysis to identify key determinants within this group of variables that could influence both knowledge and practice of healthy behaviors. To examine this, we used centrality matrices, which quantify the importance of each variable within the network.

*Centrality Matrices*

Centrality measures help determine which variables are most influential in the network by assessing their position and connectivity relative to others based on a correlation matrix^8^. The three centrality parameters were considered when interpreting the results: (i) Degree centrality measures how many direct connections a variable has with others. A variable with high degree centrality is directly related to many others, reflecting the number of significant relationships. (ii) Closeness centrality measures how efficiently a variable can reach all others in the network, both directly and indirectly. A variable with high closeness centrality has shorter average paths to other variables, meaning it can influence or be influenced by them more quickly, with fewer intermediate steps. (iii) Betweenness centrality reflects the extent to which a variable acts as a bridge between different parts of the network. A high betweenness variable connects otherwise separate groups, highlighting potential intervention targets.

**Interpretation of the network analysis figures**

*Figure 3 Correlation Network.*

This network plot visually represents the relationships between knowledge, practice, facilitators, barriers, and demographics. Nodes (circles) represent individual variables. Edges (lines between nodes) indicate correlations, with thicker lines representing stronger correlations^7^. Highly connected nodes are positioned at the center (high degree centrality), indicating key determinants with strong correlations, while peripheral nodes have fewer connections and represent less influential factors. Bridging nodes (high betweenness centrality) link different clusters, highlighting potential intervention targets, and closely grouped nodes suggest variables that frequently co-occur.

**References**

1. Guess MJ, Wilson SB. Introduction to Hierarchical Clustering: Journal of Clinical Neurophysiology. 2002;19(2):144-151.

2. Liang X, Sha Q, Rho Y, Zhang S. A hierarchical clustering method for dimension reduction in joint analysis of multiple phenotypes. Genet Epidemiol. 2018;42(4):344-353.

3. Austin RR, McLane TM, Pieczkiewicz DS, Adam T, Monsen KA. Advantages and disadvantages of using theory-based versus data-driven models with social and behavioral determinants of health data. Journal of the American Medical Informatics Association. 2023;30(11):1818-1825.

4. Ranganathan P, Pramesh CS, Buyse M. Common pitfalls in statistical analysis: The perils of multiple testing. Perspect Clin Res. 2016;7(2):106-107.

5. Zhang Z, Murtagh F, Van Poucke S, Lin S, Lan P. Hierarchical cluster analysis in clinical research with heterogeneous study population: highlighting its visualization with R. Ann Transl Med. 2017;5(4):75.

6. Saary MJ. Radar plots: a useful way for presenting multivariate health care data. Journal of Clinical Epidemiology. 2008;61(4):311-317.

7. Costantini G, Epskamp S, Borsboom D, et al. State of the aRt personality research: A tutorial on network analysis of personality data in R. Journal of Research in Personality. 2015;54:13-29.

8. Rodebaugh TL, Tonge NA, Piccirillo ML, et al. Does centrality in a cross-sectional network suggest intervention targets for social anxiety disorder? J Consult Clin Psychol. 2018;86(10):831-844.

# Tables

## Table S1: HOBCAS Questionnaire

| **No.** | **Question** | | | **Options** | **Question used in this study** |
| --- | --- | --- | --- | --- | --- |
|  | Please enter your unique Prolific ID | | | Open question | Yes |
|  | Please enter your 5-number zip code | | | Open question | No |
|  | Please enter your age | | | Open Question | Yes |
|  | Please select the race/ethnicity with which you identify | | | a. White  b. Black / African American  c. American Indian / Alaskan Native  d. Hispanic / Latino / Spanish Origin  e. Chinese  f. Filipino  g. Asian Indian  h. Other Asian  i. Vietnamese  j. Korean  k. Japanese  l. Native Hawaiian  m. Samoan  n. Chamorro  o. Other Pacific Islander  p. Other | Yes |
|  | What was your biological sex assigned at birth? | | | a. Female  b. Male  c. Intersex  d. None of the above describe me  e. Prefer not to answer  f. Unknown | Yes |
|  | Please select the highest level of education you have completed | | | a. No schooling completed  b. Nursery-Kindergarten  c. Grade 1-12: No diploma  d. High School diploma, GED or alternative credential  e. Associate’s degree or bachelor’s degree  f. Graduate degree | Yes |
|  | Which of the following categories best describes your marital status | | | a. Currently married  b. Divorced  c. Separated  d. Widowed  e. Never married | Yes |
|  | Which of the following categories best describes your employment status? | | | a. Full-time employment  b. Part-time employment  c. Self-employed  d. Student  e. Full-time caregiver  f. Not employed, looking for work  g. Not employed, NOT looking for work  h. Retired  i. Unable to work | Yes |
|  | What is your living situation today? | | | a. I have a steady place to live  b. I have a steady place to live today,   but am worried about losing it in the future  c. I do not have a steady place to live | No |
|  | Which of the following best describes your living status? | | | a. Homeowner  b. Renting  c. Occupying without payment of cash rent  d. Other | No |
|  | Total household income: how much total combined money did all members of your household earn in 2022? This includes money from jobs, net income from businesses, farms or rent; pensions; dividends; interest; social security payments and any other income received by members of your household. Please report the total amount of money earned - do not subtract the amount you paid in taxes or any deductions listed on your tax returns. | | | a. $0-$20,550  b. $20,551-$83,550  c. $83,551-$178,150  d. $178,151-$340,100  e. $340,101-$431,900  f. $431,901-$647,850  g. $647,851 or more | No |
|  | Total personal income: how much money did you earn personally in 2022? This includes money from jobs, net income from businesses, farms or rent; pensions; dividends; interest; social security payments and any other income received by you. Please report the total amount of money earned - do not subtract the amount you paid in taxes or any deductions listed on your tax returns. | | | a. $0-$10,275  b. $10,276-$41,775  c. $41,776-$89,075  d. $89,076-$170,050  e. $170,051-$215,950  f. $215,951-$539,900  g. $539,001 or more | Yes |
|  | Have you ever known someone who has had a diagnosis of dementia or stroke? | | | a. Yes  b. No | Yes |
|  | Have you ever been a caregiver for someone who has had a diagnosis of dementia or stroke? | | | a. Yes  b. No | Yes |
|  | Dementia is a normal part of the ageing process | | | 5-point Likert scale: (i) strongly disagree, (ii) somewhat disagree, (iii) not sure, (iv) somewhat agree or (v) strongly agree | No |
|  | Alzheimer's disease is the most common form of dementia | | | 5-point Likert scale: (i) strongly disagree, (ii) somewhat disagree, (iii) not sure, (iv) somewhat agree or (v) strongly agree | No |
|  | Dementia cannot be prevented by changing lifestyle behavior | | | 5-point Likert scale: (i) strongly disagree, (ii) somewhat disagree, (iii) not sure, (iv) somewhat agree or (v) strongly agree | No |
|  | Having a high blood pressure does not increase a person's risk of developing dementia | | | 5-point Likert scale: (i) strongly disagree, (ii) somewhat disagree, (iii) not sure, (iv) somewhat agree or (v) strongly agree | No |
|  | Dementia can be diagnosed at very early stages | | | 5-point Likert scale: (i) strongly disagree, (ii) somewhat disagree, (iii) not sure, (iv) somewhat agree or (v) strongly agree | No |
|  | Dementia can be caused by blood vessel disease | | | 5-point Likert scale: (i) strongly disagree, (ii) somewhat disagree, (iii) not sure, (iv) somewhat agree or (v) strongly agree | No |
|  | Maintaining a healthy lifestyle reduces the risk of developing dementia | | | 5-point Likert scale: (i) strongly disagree, (ii) somewhat disagree, (iii) not sure, (iv) somewhat agree or (v) strongly agree | No |
|  | Exercise is generally beneficial for reducing the risk of dementia | | | 5-point Likert scale: (i) strongly disagree, (ii) somewhat disagree, (iii) not sure, (iv) somewhat agree or (v) strongly agree | No |
|  | Medications are the most effective way to reduce the risk of developing dementia | | | 5-point Likert scale: (i) strongly disagree, (ii) somewhat disagree, (iii) not sure, (iv) somewhat agree or (v) strongly agree | No |
|  | Smoking is a risk factor for stroke and dementia | | | 5-point Likert scale: (i) strongly disagree, (ii) somewhat disagree, (iii) not sure, (iv) somewhat agree or (v) strongly agree | No |
|  | Stroke is more common in women | | | 5-point Likert scale: (i) strongly disagree, (ii) somewhat disagree, (iii) not sure, (iv) somewhat agree or (v) strongly agree | No |
|  | Having a stroke increases a person's risk of having a second stroke at a later time | | | 5-point Likert scale: (i) strongly disagree, (ii) somewhat disagree, (iii) not sure, (iv) somewhat agree or (v) strongly agree | No |
|  | Maintaining a healthy lifestyle does not reduce a person's risk of stroke | | | 5-point Likert scale: (i) strongly disagree, (ii) somewhat disagree, (iii) not sure, (iv) somewhat agree or (v) strongly agree | No |
|  | Having a high cholesterol does not increase your risk of stroke | | | 5-point Likert scale: (i) strongly disagree, (ii) somewhat disagree, (iii) not sure, (iv) somewhat agree or (v) strongly agree | No |
|  | Drinking small amounts of alcohol (a glass of wine a day) is protective against stroke and dementia | | | 5-point Likert scale: (i) strongly disagree, (ii) somewhat disagree, (iii) not sure, (iv) somewhat agree or (v) strongly agree | No |
|  | Less than 7 hours of sleep, or an untreated sleep disorder can be a risk factor for dementia | | | 5-point Likert scale: (i) strongly disagree, (ii) somewhat disagree, (iii) not sure, (iv) somewhat agree or (v) strongly agree | No |
|  | I feel at high risk of developing dementia | | | 5-point Likert scale: (i) strongly disagree, (ii) somewhat disagree, (iii) not sure, (iv) somewhat agree or (v) strongly agree | No |
|  | I feel at high risk of having a stroke | | | 5-point Likert scale: (i) strongly disagree, (ii) somewhat disagree, (iii) not sure, (iv) somewhat agree or (v) strongly agree | No |
|  | Information and advice from medical professionals can help me reduce my risk of developing dementia | | | 5-point Likert scale: (i) strongly disagree, (ii) somewhat disagree, (iii) not sure, (iv) somewhat agree or (v) strongly agree | No |
|  | Information and advice from medical professionals can help me reduce my risk of having a stroke | | | 5-point Likert scale: (i) strongly disagree, (ii) somewhat disagree, (iii) not sure, (iv) somewhat agree or (v) strongly agree | No |
|  | Changing lifestyle and health habits can help reduce my risk of developing dementia | | | 5-point Likert scale: (i) strongly disagree, (ii) somewhat disagree, (iii) not sure, (iv) somewhat agree or (v) strongly agree | No |
|  | Changing lifestyle and health habits can help reduce my risk of having a stroke | | | 5-point Likert scale: (i) strongly disagree, (ii) somewhat disagree, (iii) not sure, (iv) somewhat agree or (v) strongly agree | No |
|  | Changing lifestyle and health habits is difficult to maintain over a long period of time | | | 5-point Likert scale: (i) strongly disagree, (ii) somewhat disagree, (iii) not sure, (iv) somewhat agree or (v) strongly agree | No |
|  | I am too busy to change my lifestyle and health habits | | | 5-point Likert scale: (i) strongly disagree, (ii) somewhat disagree, (iii) not sure, (iv) somewhat agree or (v) strongly agree | No |
|  | My financial situation does not allow me to change my lifestyle and behavior | | | 5-point Likert scale: (i) strongly disagree, (ii) somewhat disagree, (iii) not sure, (iv) somewhat agree or (v) strongly agree | No |
|  | Learning more about dementia and stroke motivates me to change my lifestyle | | | 5-point Likert scale: (i) strongly disagree, (ii) somewhat disagree, (iii) not sure, (iv) somewhat agree or (v) strongly agree | No |
|  | Having risk factors for dementia and stroke makes me want to change my lifestyle | | | 5-point Likert scale: (i) strongly disagree, (ii) somewhat disagree, (iii) not sure, (iv) somewhat agree or (v) strongly agree | No |
|  | I am confident that I can change my lifestyle and behaviour so I can reduce the risk of developing dementia and stroke | | | 5-point Likert scale: (i) strongly disagree, (ii) somewhat disagree, (iii) not sure, (iv) somewhat agree or (v) strongly agree | No |
|  | To what extent do you believe these factors contribute to making your brain healthy? | Maintaining a healthy blood pressure | | 5-point Likert scale: (i) very useful, (ii) somewhat useful, (iii) not sure, (iv) not very useful, (v) not useful at all | No |
| **43.** |  | Maintaining healthy blood sugar levels | | 5-point Likert scale: (i) very useful, (ii) somewhat useful, (iii) not sure, (iv) not very useful, (v) not useful at all | No |
| **43.** |  | Maintaining healthy levels of cholesterol | | 5-point Likert scale: (i) very useful, (ii) somewhat useful, (iii) not sure, (iv) not very useful, (v) not useful at all | No |
| **43.** |  | Maintaining a healthy weight | | 5-point Likert scale: (i) very useful, (ii) somewhat useful, (iii) not sure, (iv) not very useful, (v) not useful at all | No |
| **43.** |  | Maintaining a healthy diet | | 5-point Likert scale: (i) very useful, (ii) somewhat useful, (iii) not sure, (iv) not very useful, (v) not useful at all | Yes |
| **43.** |  | Low alcohol intake | | 5-point Likert scale: (i) very useful, (ii) somewhat useful, (iii) not sure, (iv) not very useful, (v) not useful at all | Yes |
| **43.** |  | Not smoking | | 5-point Likert scale: (i) very useful, (ii) somewhat useful, (iii) not sure, (iv) not very useful, (v) not useful at all | Yes |
| **43.** |  | Consistent physical activity | | 5-point Likert scale: (i) very useful, (ii) somewhat useful, (iii) not sure, (iv) not very useful, (v) not useful at all | Yes |
| **43.** |  | A healthy sleep schedule | | 5-point Likert scale: (i) very useful, (ii) somewhat useful, (iii) not sure, (iv) not very useful, (v) not useful at all | Yes |
| **43.** |  | Managing levels of stress | | 5-point Likert scale: (i) very useful, (ii) somewhat useful, (iii) not sure, (iv) not very useful, (v) not useful at all | Yes |
| **43.** |  | Maintaining close social relationships | | 5-point Likert scale: (i) very useful, (ii) somewhat useful, (iii) not sure, (iv) not very useful, (v) not useful at all | Yes |
| **43.** |  | Finding purpose / meaning in life | | 5-point Likert scale: (i) very useful, (ii) somewhat useful, (iii) not sure, (iv) not very useful, (v) not useful at all | Yes |
| **43.** |  | Brain training apps and games | | 5-point Likert scale: (i) very useful, (ii) somewhat useful, (iii) not sure, (iv) not very useful, (v) not useful at all | No |
| **43.** |  | Brain supplements (fish oil, etc.) | | 5-point Likert scale: (i) very useful, (ii) somewhat useful, (iii) not sure, (iv) not very useful, (v) not useful at all | No |
| **43.** |  | Practicing meditation and mindfulness techniques | | 5-point Likert scale: (i) very useful, (ii) somewhat useful, (iii) not sure, (iv) not very useful, (v) not useful at all | No |
| **44.** | Which of these factors do you believe are the most difficult to change/maintain in daily life? | Maintaining a healthy blood pressure | | 5-point Likert scale: (i) very useful, (ii) somewhat useful, (iii) not sure, (iv) not very useful, (v) not useful at all | No |
| **44.** |  | Maintaining healthy blood sugar levels | | 5-point Likert scale: (i) very useful, (ii) somewhat useful, (iii) not sure, (iv) not very useful, (v) not useful at all | No |
| **44.** |  | Maintaining healthy levels of cholesterol | | 5-point Likert scale: (i) very useful, (ii) somewhat useful, (iii) not sure, (iv) not very useful, (v) not useful at all | No |
| **44.** |  | Maintaining a healthy weight | | 5-point Likert scale: (i) very useful, (ii) somewhat useful, (iii) not sure, (iv) not very useful, (v) not useful at all | No |
| **44.** |  | Maintaining a healthy diet | | 5-point Likert scale: (i) very useful, (ii) somewhat useful, (iii) not sure, (iv) not very useful, (v) not useful at all | No |
| **44.** |  | Low alcohol intake | | 5-point Likert scale: (i) very useful, (ii) somewhat useful, (iii) not sure, (iv) not very useful, (v) not useful at all | No |
| **44.** |  | Not smoking | | 5-point Likert scale: (i) very useful, (ii) somewhat useful, (iii) not sure, (iv) not very useful, (v) not useful at all | No |
| **44.** |  | Consistent physical activity | | 5-point Likert scale: (i) very useful, (ii) somewhat useful, (iii) not sure, (iv) not very useful, (v) not useful at all | No |
| **44.** |  | A healthy sleep schedule | | 5-point Likert scale: (i) very useful, (ii) somewhat useful, (iii) not sure, (iv) not very useful, (v) not useful at all | No |
| **44.** |  | Managing levels of stress | | 5-point Likert scale: (i) very useful, (ii) somewhat useful, (iii) not sure, (iv) not very useful, (v) not useful at all | No |
| **44.** |  | Maintaining close social relationships | | 5-point Likert scale: (i) very useful, (ii) somewhat useful, (iii) not sure, (iv) not very useful, (v) not useful at all | No |
| **44.** |  | Finding purpose / meaning in life | | 5-point Likert scale: (i) very useful, (ii) somewhat useful, (iii) not sure, (iv) not very useful, (v) not useful at all | No |
| **44.** |  | Brain training apps and games | | 5-point Likert scale: (i) very useful, (ii) somewhat useful, (iii) not sure, (iv) not very useful, (v) not useful at all | No |
| **44.** |  | Brain supplements (fish oil, etc.) | | 5-point Likert scale: (i) very useful, (ii) somewhat useful, (iii) not sure, (iv) not very useful, (v) not useful at all | No |
| **44.** |  | Practicing meditation and mindfulness techniques | | 5-point Likert scale: (i) very useful, (ii) somewhat useful, (iii) not sure, (iv) not very useful, (v) not useful at all | No |
| **45.** | If you had to pick only one, which of the following factors do you believe has the greatest impact on keeping your brain healthy? | | | a. Maintaining a healthy blood pressure  b. Maintaining healthy blood sugar levels  c. Maintaining healthy levels of cholesterol  d. Maintaining a healthy weight  e. Maintaining a healthy diet  f. Low alcohol intake  g. Not smoking  h. Consistent physical activity  i. A healthy sleep schedule  j. Managing levels of stress  k. Maintaining close social relationships  l. Finding purpose / meaning in life | No |
| **46** | Please rank the following factors based on which you would be most likely to change in your own daily life | | | a. Maintaining a healthy blood pressure  b. Maintaining healthy blood sugar levels  c. Maintaining healthy levels of cholesterol  d. Maintaining a healthy weight  e. Maintaining a healthy diet  f. Low alcohol intake  g. Not smoking  h. Consistent physical activity  i. A healthy sleep schedule  j. Managing levels of stress  k. Maintaining close social relationships  l. Finding purpose / meaning in life | No |
| **47** | Have you ever been diagnosed with hypertension (high blood pressure)? If you know your most recent blood pressure measurement, please enter it below using the "other" text option | | | a. Yes  b. No  c. Other | No |
| **48** | Have you ever been diagnosed with diabetes? If you know your most recent Hemoglobin A1c (HbA1c) measurement, please enter it below using the "other" text option | | | a. Yes, I have a diagnosis of diabetes  b. I have a diagnosis of prediabetes (impaired glucose tolerance)  c. No  d. Other | No |
| **49** | Have you ever been diagnosed with hypercholesterolemia (high cholesterol)? | | | a. Yes, and I am on medication  b. Yes, but I am not on medication  c. no | No |
| **50** | Please enter your current weight in pounds and ounces (lbs and oz) | | |  | No |
| **51** | Please enter your height in feet and inches (ft and in) | | |  | No |
| **52** | Which of the following do you include in your typical diet? | | | a. 4.5 servings of fruit and vegetables per day  b. 2 servings of lean protein per day  c. 3 or more servings of whole grains per day  d. Less than 1,5 grams of sodium per day  e. Less than 36oz of sugar sweet beverages (soda, juice etc.) per week | Yes |
| **53** | How many alcoholic drinks do you consume per week? | | | a. 0-1 alcoholic drinks per week  b. 2-3 alcoholic drinks per week  c. 4 or more alcoholic drinks per week | Yes |
| **54** | Do you smoke? | | | a. Current smoker  b. Quit within the last year  c. Quit more than one year ago  d. Never smoked | Yes |
| **55** | How much aerobic exercise do you do per week? | | | a. Less than 150 minutes of moderate, or less than 75 minutes of high intensity physical activity per week  b. At least 150 minutes of moderate, or more than 75 minutes of high intensity physical activity per week | Yes |
| **56** | How long do you sleep every night? | | | a. Less than 6 hours per night or sleep problems  b. 6-8 hours per night without a sleep problem  c. More than 8 hours per night or sleep problems | Yes |
| **57** | How would you describe your daily level of stress? | | | a. High level of stress than often makes it difficult to function  b. Moderate level of stress that occasionally makes it difficult to function  c. Manageable level of stress that rarely makes it difficult to function | Yes |
| **58** | How many close social connections do you have? Close social connections are people other than your partner or children, that you feel close with and could talk about private matters or call upon for help. | | | a. I have few or no close connections other than my partner or children  b. I have at least two people, other than my partner or children, that I feel close with and could talk about private matters or call upon for help | Yes |
| **59** | Do you often struggle to find meaning in life? | | | a. I often struggle to find value or purpose in my life  b. I generally feel that my life has meaning and/or purpose | Yes |
| **60** | Who do you trust when you are looking for information about your health? | | Primary care physicians | 5-point Likert scale: (i) strongly trust, (ii) somewhat trust(iii) not sure, (iv) somewhat do not trust (v) strongly do not trust | No |
| **60** |  |  | Specialist physicians | 5-point Likert scale: (i) strongly trust, (ii) somewhat trust(iii) not sure, (iv) somewhat do not trust (v) strongly do not trust | No |
| **60** |  |  | Other healthcare professionals (nurses, NP's) | 5-point Likert scale: (i) strongly trust, (ii) somewhat trust(iii) not sure, (iv) somewhat do not trust (v) strongly do not trust | No |
| **60** |  |  | Religious organizations and leaders | 5-point Likert scale: (i) strongly trust, (ii) somewhat trust(iii) not sure, (iv) somewhat do not trust (v) strongly do not trust | No |
| **60** |  |  | Scientists | 5-point Likert scale: (i) strongly trust, (ii) somewhat trust(iii) not sure, (iv) somewhat do not trust (v) strongly do not trust | No |
| **60** |  |  | Podcasts, radio and television | 5-point Likert scale: (i) strongly trust, (ii) somewhat trust(iii) not sure, (iv) somewhat do not trust (v) strongly do not trust | No |
| **60** |  |  | Family and friends | 5-point Likert scale: (i) strongly trust, (ii) somewhat trust(iii) not sure, (iv) somewhat do not trust (v) strongly do not trust | No |
| **60** |  |  | Social media | 5-point Likert scale: (i) strongly trust, (ii) somewhat trust(iii) not sure, (iv) somewhat do not trust (v) strongly do not trust | No |
| **60** |  |  | Newspapers and magazines (including online editions) | 5-point Likert scale: (i) strongly trust, (ii) somewhat trust(iii) not sure, (iv) somewhat do not trust (v) strongly do not trust | No |
| **60** |  |  | Websites, or self-search (Google) | 5-point Likert scale: (i) strongly trust, (ii) somewhat trust(iii) not sure, (iv) somewhat do not trust (v) strongly do not trust | No |
| **60** |  |  | Politicians | 5-point Likert scale: (i) strongly trust, (ii) somewhat trust(iii) not sure, (iv) somewhat do not trust (v) strongly do not trust | No |
| **60** |  |  | Government health agencies | 5-point Likert scale: (i) strongly trust, (ii) somewhat trust(iii) not sure, (iv) somewhat do not trust (v) strongly do not trust | No |
| **60** |  |  | Charitable organizations | 5-point Likert scale: (i) strongly trust, (ii) somewhat trust(iii) not sure, (iv) somewhat do not trust (v) strongly do not trust | No |
| **61** | Did you understand all of the questions you were asked in this survey? If no, please write down the question numbers below. | | | Open | Yes |
| **62** | Would you have preferred to take this survey in a language other than English? | | | Open | Yes |

## Table S2: Comparing Cohort to U.S. Census data.

| **Demographics** | | **Included cohort N=1478** | **2023 U.S. Census Data N=334,914,896** | **P value** |
| --- | --- | --- | --- | --- |
| Age, mean (SD) | | 45.5 (15.9) | 39.2 (0.1) | <0.001 |
| Female, N (%) | | 754 (51.8) | 169,185,523 (50.5) | 0.16 |
| Ethnicity N (%) | White | 1168 (79.0)* | 252,190,916,688 (75.3)* | <0.001 |
|  | Black/ African American | 192 (13.1) | 45,883,340,752 (13.7) |  |
|  | Asian | 91 (6.2) | 21,434,553,344 (6.4) |  |
|  | Other | 17 (1.2)* | 15,406,085,216 (4.6)* |  |
| Education,  N (%) | High school diploma or less | 453 (31.0)* | 98,418,487 (37.5)* | <0.001 |
|  | Associates degree | 713 (48.8) | 130,599,763 (49.8) |  |
|  | Graduate Degree | 295 (20.2)* | 33,248,210 (12.7)* |  |
| Marital status,   N (%) | Never married | 611 (42.6)* | 94,798,794,160 (34.4)* | <0.001 |
|  | Currently married | 603 (42.0)* | 132,552,965,090 (48.1)* |  |
|  | Separated, divorced, widowed | 221 (15.4)* | 48,226,130,750 (17.5)* |  |
| Employment,   N (%) | Employed | 1008 (69.9)* | 164,346,993 (60.9%)* | <0.001 |
|  | Unemployed | 156 (10.8)* | 7,395,929 (2.7%)* |  |
|  | Not in labor force | 278 (19.3)* | 98,083,934 (36.4)* |  |

Legend. Table S2. Denotes the number of participants. % represents proportions. * Indicates statistical significance in post-hoc pairwise comparison.

## Table S3: Jaccard bootstrap

|  | Cluster 1 | Cluster 2 | Cluster 3 |
| --- | --- | --- | --- |
| Clusterwise Jaccard bootstrap mean | 0.60 | 0.71 | 0.73 |
| Dissolved clusters | 29 times | 0 times | 15 times |
| Recovered clusters | 15 times | 34 times | 57 times |

Legend. Table S3 – Jaccard bootstrap.

## Table S4: Distribution of knowledge and practice of healthy behavior

| **Risk factor** | **Cluster 1**  High knowledge  Poor practice | **Cluster 2**  High knowledge  Good practice | **Cluster 3**  Lower knowledge  Poor practice | **P-value** |
| --- | --- | --- | --- | --- |
| **Knowing that a risk factor is important to keep the brain healthy** | | | | |
| Alcohol use | 0.90^3^ | 0.90^3^ | 0.66^1.2^ | <0.001 |
| Healthy diet | 0.99^3^ | 0.98^3^ | 0.85^1.2^ | <0.001 |
| Smoking | 0.97^3^ | 0.98^3^ | 0.82^1.2^ | <0.001 |
| Consistent Physical activity | 0.98^3^ | 0.97^3^ | 0.83^1.2^ | <0.001 |
| Regular Sleep schedule | 0.98^3^ | 0.98^3^ | 0.86^1.2^ | <0.001 |
| Manageable Stress | 0.98^3^ | 0.97^3^ | 0.83^1.2^ | <0.001 |
| Presence of social relationship | 0.87^3^ | 0.88^3^ | 0.06^1.2^ | <0.001 |
| Purpose in life | 0.85^3^ | 0.87^3^ | 0.07^1.2^ | <0.001 |
| **Practicing healthy behavior** | | | | |
| Alcohol use | 0.83^2,3^ | 0.71^1^ | 0.74^1^ | <0.001 |
| Healthy diet | 0.30^2^ | 0.51^1,3^ | 0.23^2^ | <0.001 |
| Smoking | 0.84 | 0.83 | 0.78 | 0.11 |
| Consistent Physical activity | 0.13^2,3^ | 0.49^1,3^ | 0.23^1,2^ | <0.001 |
| Regular Sleep schedule | 0.33 | 0.34 | 0.29 | 0.29 |
| Manageable Stress | 0.18^2,3^ | 0.57^1,3^ | 0.39^1,2^ | <0.001 |
| Presence of social relationship | 0.21^2,3^ | 0.80^1,3^ | 0.60^1,2^ | <0.001 |
| Purpose in life | 0.25^2,3^ | 0.82^1,3^ | 0.53^1,2^ | <0.001 |

Legend Table S4. Distribution of perceptions and practices across clusters. All values are presented as proportions. Superscript numbers indicate statistically significant differences (p < 0.05) between the corresponding cluster.

## Table S5: Sensitivity analysis: distribution of hierarchical clustering and k means clustering.

|  | Hierarchical clustering method | | | K means clustering method | | |
| --- | --- | --- | --- | --- | --- | --- |
|  | Cluster 1 | Cluster 2 | Cluster 3 | Cluster 1 | Cluster 2 | Cluster 3 |
| Variable | N=280 | N=825 | N=287 | N=511 | N=521 | N=360 |
| proportion knowing that a risk factor is important to keep the brain healthy | | | | | | |
| Alcohol | 0.90 | 0.90 | 0.66 | 0.91 | 0.93 | 0.66 |
| Physical activity | 0.98 | 0.97 | 0.83 | 0.98 | 0.99 | 0.83 |
| Diet | 0.99 | 0.98 | 0.85 | 0.98 | 0.99 | 0.87 |
| Stress | 0.98 | 0.97 | 0.84 | 0.99 | 0.98 | 0.83 |
| Smoking | 0.97 | 0.97 | 0.82 | 0.97 | 0.99 | 0.83 |
| Social Relationships | 0.87 | 0.88 | 0.06 | 0.91 | 0.94 | 0.09 |
| Sleep | 0.98 | 0.98 | 0.86 | 0.98 | 0.99 | 0.85 |
| Purpose in life | 0.85 | 0.87 | 0.07 | 0.91 | 0.91 | 0.11 |
| Proportion practicing healthy behavior | | | | | | |
| Alcohol | 0.83 | 0.71 | 0.74 | 0.75 | 0.72 | 0.74 |
| Stress | 0.18 | 0.57 | 0.39 | 0.08 | 0.83 | 0.43 |
| Diet | 0.30 | 0.51 | 0.24 | 0.27 | 0.67 | 0.24 |
| Purpose in life | 0.25 | 0.82 | 0.53 | 0.46 | 0.92 | 0.53 |
| Physical activity | 0.13 | 0.49 | 0.23 | 0.24 | 0.56 | 0.27 |
| Sleep | 0.33 | 0.34 | 0.29 | 0.20 | 0. 47985 | 0.26 |
| Smoking | 0.84 | 0.83 | 0.78 | 0.80 | 0.87 | 0.77 |
| Social Relationship | 0.21 | 0.80 | 0.60 | 0.48 | 0.86 | 0.54 |

Legend Table S5. Sensitivity analysis means of hierarchical clustering methods and k means clustering methods. N denotes the number.

# Figures

## Figure S1: Flowchart


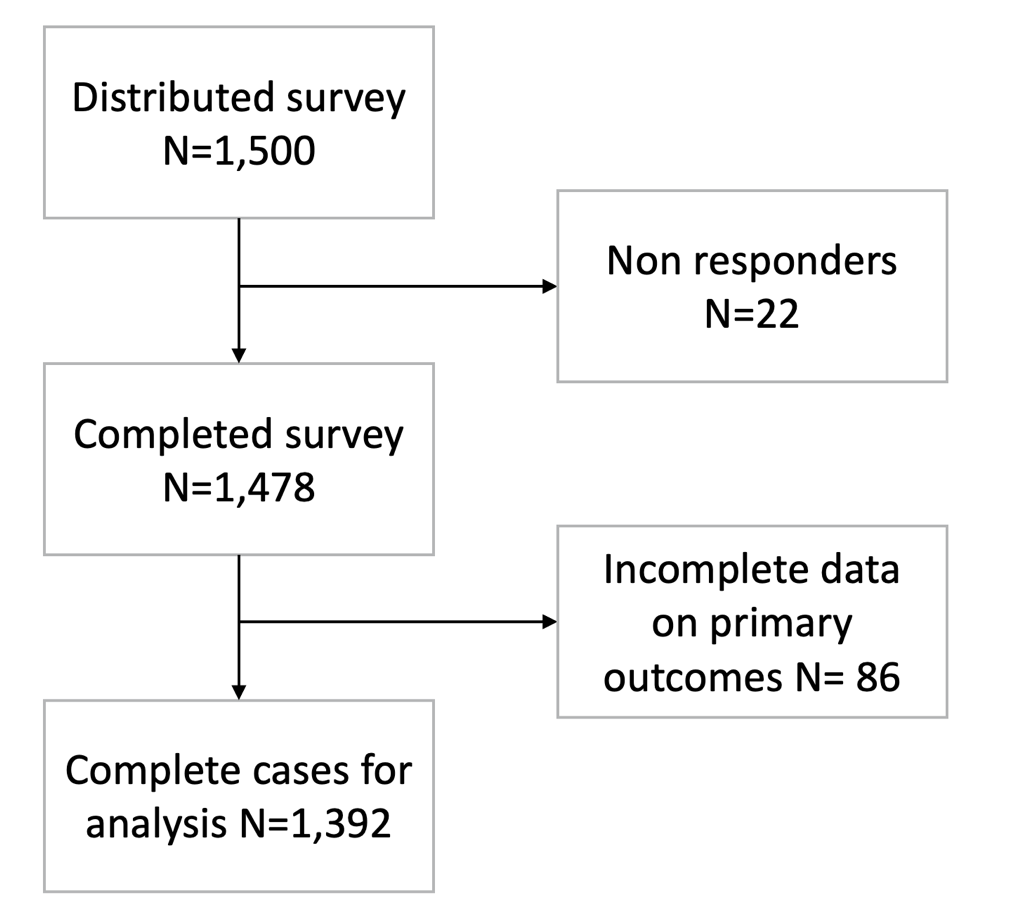


Legend S1. Flowchart – overview of all included cases for analysis. N denotes the number of participants.

## Figure S2: total-within cluster sum of squares


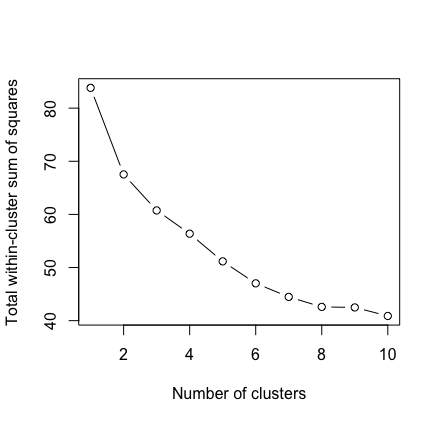


Legend. Figure S2. Total-within cluster sum of square plot. The x-asis presents the number of clusters, the y-axis present the total within-cluster sum of squares.

## Figure S3: correlation matrix


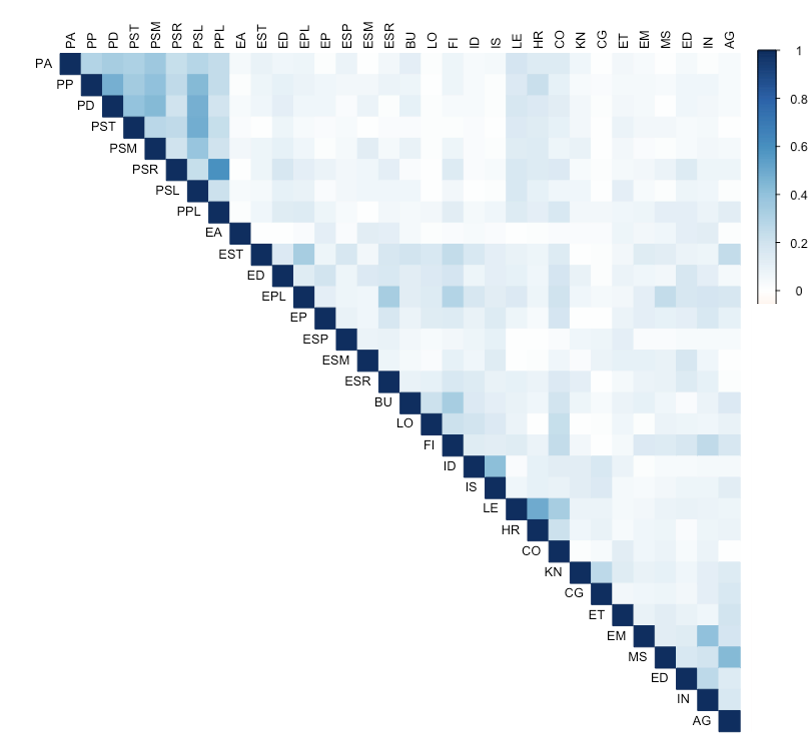


Legend. Figure S3 – Cramer V’s correlation matrix. White indicates low correlation (min 0), blue indicates a high correlation (max 1.0). PA: Knowing that low alcohol is a contributor to keeping your brain healthy, P.D.: Knowing that a healthy diet is a contributor to keeping your brain healthy, PPA.: Knowing that physical activity is a contributor to keeping your brain healthy, PPL: Knowing that purpose in life is a contributor to keeping your brain healthy, PSL: Knowing that regular sleep is a contributor to keeping your brain healthy, PSM: Knowing that not smoking is a contributor to keeping your brain healthy, PSR: Knowing that social relations are a contributor to keeping your brain healthy, PST: Knowing that manageable levels of stress are a contributor to keeping your brain healthy. Health Related E.A.: practice of healthy alcohol behavior, E.D.: practice of healthy diet behavior, EPL: having purpose in life, E.P.: practice of healthy physical activity behavior., ESP: practice of healthy sleep behavior, ESM: not smoking, ESR: Having at least two people, other than partner or children, to talk to, EST: Having manageable levels of stress. Demographics: AG: Age, E.T.: Ethnicity, E.D.: Education, MS: Marital status, EM: Employment, IN: Income. Facilitators L.E.: Learning more about dementia and stroke motivates me to change my lifestyle, I.S.: I feel at high risk of having a stroke. H.R. Having risk factors for dementia and stroke makes me want to change my lifestyle, CO: I am confident that I can change my lifestyle and behavior so I can reduce the risk of developing dementia and stroke, ID: I feel at high risk of developing dementia, L.O.: I feel at high risk of having a stroke, K.N.: I have ever known someone with dementia or stroke, C.G.: I have ever been a caregiver for someone with dementia or stroke. Barriers: F.I.: My financial situation does not allow me to change my lifestyle and behavior, L.O.: Changing lifestyle and health habits is difficult to maintain over a long period of time, B.U.: I am too busy to change my lifestyle and health habits.

## Figure S4: Centrality plots


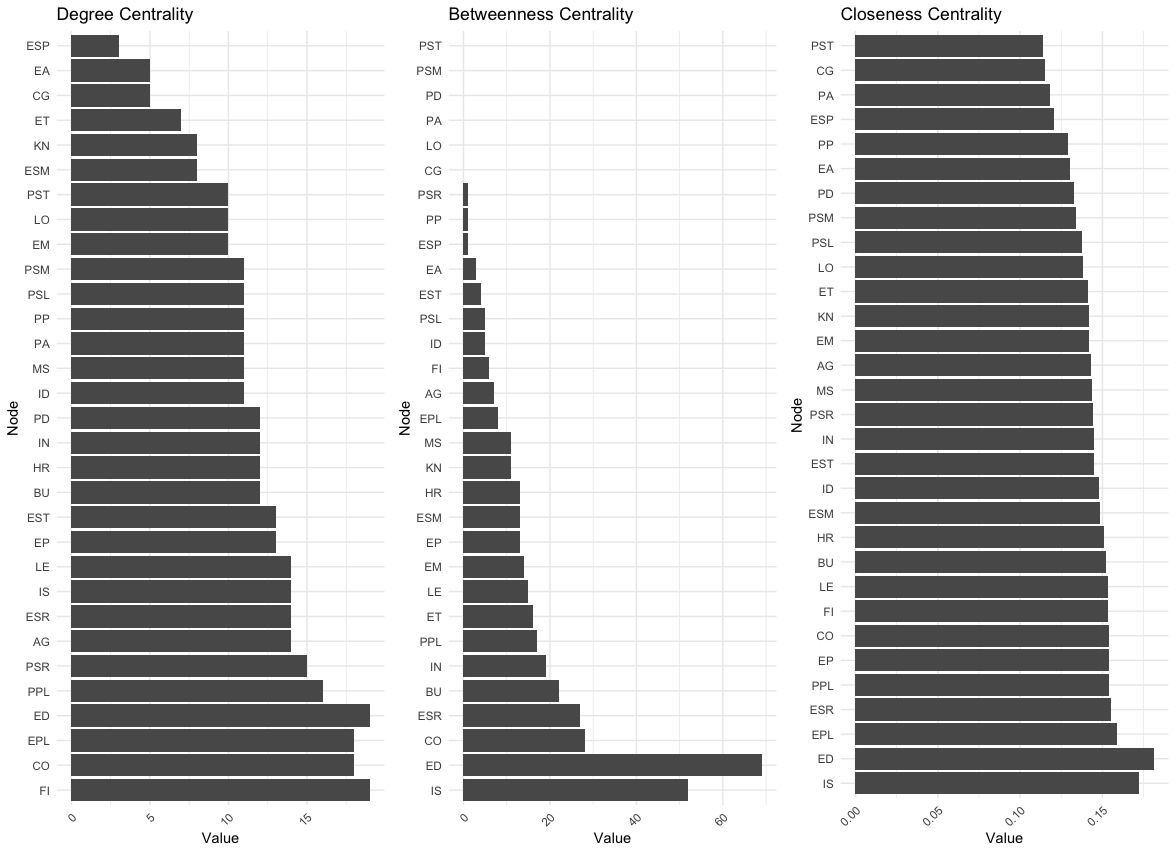


Legend S4. Centrality plot.

PA: Knowing that low alcohol is a contributor to keeping your brain healthy, P.D.: Knowing that a healthy diet is a contributor to keeping your brain healthy, PPA.: Knowing that physical activity is a contributor to keeping your brain healthy, PPL: Knowing that purpose in life is a contributor to keeping your brain healthy, PSL: Knowing that regular sleep is a contributor to keeping your brain healthy, PSM: Knowing that not smoking is a contributor to keeping your brain healthy, PSR: Knowing that social relations are a contributor to keeping your brain healthy, PST: Knowing that manageable levels of stress are a contributor to keeping your brain healthy. Health Related E.A.: practice of healthy alcohol behavior, E.D.: practice of healthy diet behavior, EPL: having purpose in life, E.P.: practice of healthy physical activity behavior., ESP: practice of healthy sleep behavior, ESM: not smoking, ESR: Having at least two people, other than partner or children, to talk to, EST: Having manageable levels of stress. Demographics: AG: Age, E.T.: Ethnicity, E.D.: Education, MS: Marital status, EM: Employment, IN: Income. Facilitators L.E.: Learning more about dementia and stroke motivates me to change my lifestyle, I.S.: I feel at high risk of having a stroke. H.R. Having risk factors for dementia and stroke makes me want to change my lifestyle, CO: I am confident that I can change my lifestyle and behavior so I can reduce the risk of developing dementia and stroke, ID: I feel at high risk of developing dementia, L.O.: I feel at high risk of having a stroke, K.N.: I have ever known someone with dementia or stroke, C.G.: I have ever been a caregiver for someone with dementia or stroke. Barriers: F.I.: My financial situation does not allow me to change my lifestyle and behavior, L.O.: Changing lifestyle and health habits is difficult to maintain over a long period of time, B.U.: I am too busy to change my lifestyle and health habits.

## Figure S5: Sensitivity analysis – differences between hierarchical and k-means cluster


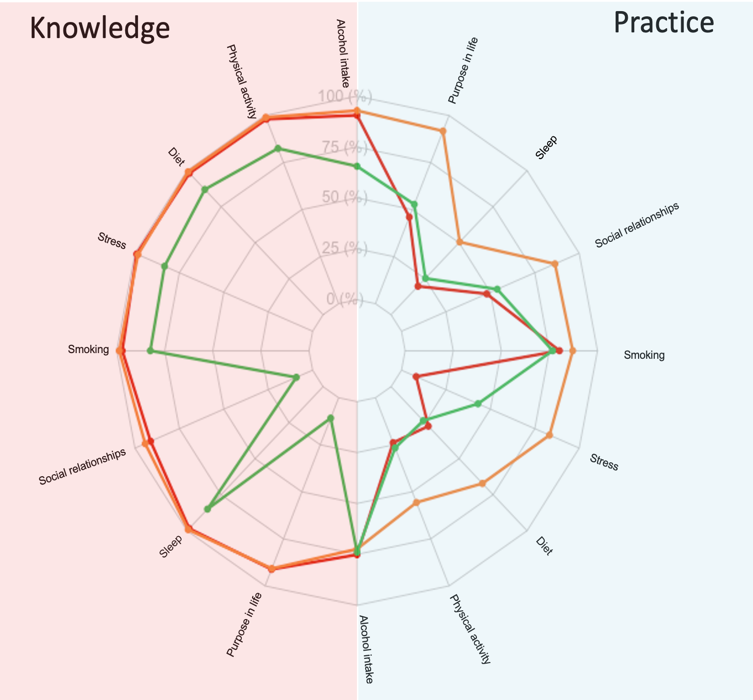
**
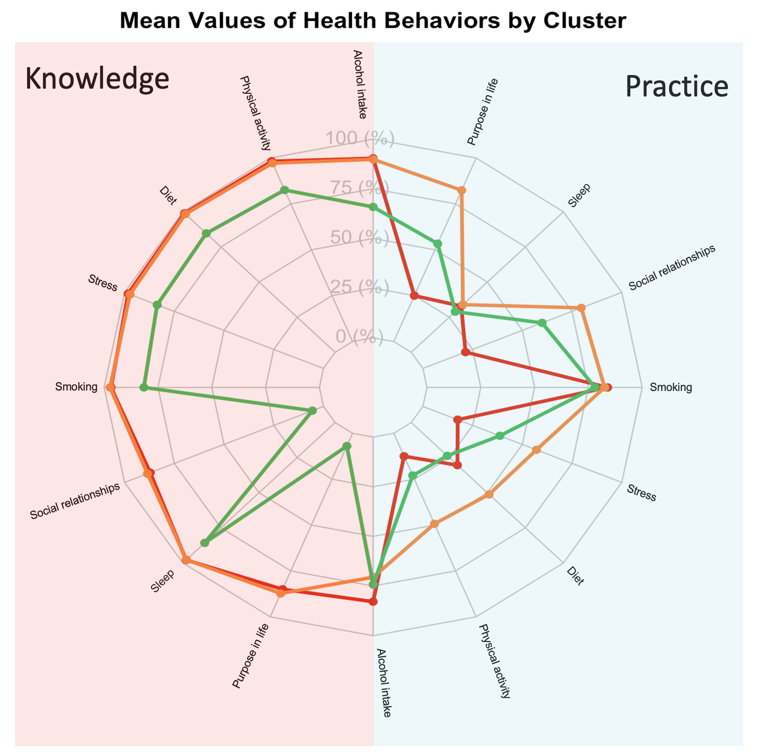
**

B

A

Legend Figure S5. The left side (pink background) of the radar plot presents the % of individuals in a cluster that have the knowledge that a certain risk factor is a useful contributor to maintaining brain health. The right side (blue background) of the plot presents the % of individuals in a cluster that practices a healthy behavior. Cluster 1 is colored red, cluster 2 is colored orange, and cluster 3 is colored green. **A.** Perceptions hierarchical clustering analysis results **B**. k-means clusters analysis results.
